# Artificial intelligence and the transformation of education: a systematic narrative review towards adaptive epistemic ecosystems and post-linear pedagogy

**DOI:** 10.3389/frai.2026.1899576

**Published:** 2026-09-11

**Authors:** Sholpan Saparbaykyzy, Zhibek Tajibayeva, Elmira Aitenova, Ayjan Nauruzbaeva, Gulnur Nakypova, Aydin Daukeeva, Timoth Mkilima

**Affiliations:** 1 Department of General Pedagogy, Faculty of Pedagogy, Yessenov University, Aktau, Kazakhstan; 2 Department of Pedagogical Psychology, Taraz Regional University named after M. Kh. Dulaty, Taraz, Kazakhstan; 3 Nukus State Pedagogical Institute named after Ajiniyaza, Nukus, Karakalpakstan, Uzbekistan; 4 Educational Program “Pedagogy, Psychology and Methods of Primary Education”, Korkyt Ata Kyzylorda State University, Kyzylorda, Kazakhstan; 5 Department of Environmental Engineering and Management, The University of Dodoma, Dodoma, Tanzania

**Keywords:** adaptive epistemic ecosystems, adaptive learning, AI governance, artificial intelligence in education, educational transformation, intelligent tutoring systems, learning analytics, systematic narrative review

## Abstract

Artificial intelligence is increasingly embedded in educational processes, yet much of the literature continues to examine individual tools rather than the relationships amongst pedagogy, curriculum, assessment, knowledge validation, institutional authority, and governance. This systematic narrative review examines these relationships whilst explicitly separating evidence-based patterns from conceptual interpretation. The review followed a PRISMA-informed protocol covering database selection, Boolean search strategy, eligibility criteria, staged screening, type-appropriate quality appraisal, structured extraction, citation auditing, and thematic synthesis. Scopus, Web of Science Core Collection, ERIC, and ScienceDirect formed the reproducible database core, whilst Google Scholar and backward and forward citation tracking were used as supplementary discovery mechanisms. After duplicate removal, title and abstract screening, full-text assessment, and quality appraisal, 124 studies were retained for synthesis. The review addressed four questions concerning how artificial intelligence is conceptualised in contemporary educational research; how it is associated with changes in pedagogy, curriculum, learning analytics, assessment, and governance; what recurring relationships connect adaptive feedback, curriculum sequencing, human-AI roles, epistemic validation, and institutional accountability; and what ethical, epistemological, and governance challenges accompany increasing human-AI participation. The synthesis indicates an uneven movement from isolated tool adoption towards more adaptive and data-informed arrangements, accompanied by substantial debate over technological determinism, platform power, teacher agency, language bias, data colonialism, and epistemic justice. Adaptive epistemic ecosystems and post-linear pedagogy are therefore advanced as synthesis-derived, integrative conceptual constructs rather than predetermined coding categories or universal empirical claims. Their contribution lies in specifying testable relationships amongst human-AI cognition, learning-data infrastructures, pedagogical adaptation, epistemic validation, governance, and contextual boundary conditions whilst preserving the distinction between observed patterns, interpretive synthesis, and future-oriented propositions.

## 1 Introduction

Education systems worldwide are confronting growing pressures associated with complexity, uncertainty, rapid technological change, and increasingly data-intensive social environments. Recent research emerging from the COVID-19 pandemic has highlighted how crises expose persistent weaknesses in conventional educational models, particularly their limited capacity to support the transfer of formal knowledge to complex real-world contexts. Wang et al. (2025a) demonstrated that despite years of formal mathematics education, many citizens relied primarily on informal reasoning rather than formal mathematical concepts when interpreting pandemic-related data, revealing a disconnect between school-based knowledge and societal problem-solving. Similar concerns have been documented in citizenship and mathematics education research (Maass et al., 2022; Makar et al., 2023; Povey and Adams, 2021), where the ability to critically interpret data, uncertainty, and predictive models emerged as an essential competency for informed participation in contemporary society. These findings indicate that educational effectiveness is no longer evaluated only in terms of content coverage or standardised performance outcomes; it is also judged by learners’ capacity to apply knowledge in dynamic and unpredictable contexts.

These challenges have coincided with the rapid expansion of artificial intelligence (AI) across educational systems. AI technologies are increasingly integrated into assessment, admissions, student support, curriculum delivery, learning analytics, and institutional decision-making processes. The COVID-19 pandemic accelerated the adoption of technology-mediated educational practices, including remote assessment, adaptive evaluation policies, and data-informed admissions procedures (Camara and Mattern, 2022). More recently, AI-enabled systems have expanded the capacity of institutions to monitor learner progress, personalise educational experiences, and generate predictive insights regarding student outcomes (Wang, 2025). Whilst these developments have often been discussed in terms of efficiency, effectiveness, or personalisation, they also raise broader questions about how educational authority, knowledge production, and decision-making are being reconfigured within increasingly algorithmic environments.

At the pedagogical level, several strands of evidence challenge the assumption that all learning is best organised through fixed and sequential instructional pathways. A systematic review in team-invasion ball sports by Bromilow et al. (2025) found context-specific advantages of nonlinear pedagogical approaches for adaptive decision-making, problem-solving, and contextual responsiveness, whilst also showing that effects depend on implementation and learning context. Similar findings have emerged across science, mathematics, and technology education, where intelligent tutoring systems and adaptive learning environments have demonstrated positive effects on learner engagement, feedback quality, and learning outcomes (Villegas-Ch et al., 2025). These developments suggest that educational processes are becoming increasingly characterised by iteration, feedback, adaptation, and contextual responsiveness rather than predetermined instructional sequences. However, the extent to which these developments constitute incremental improvement or more substantial educational transformation remains an open question requiring careful examination.

At the same time, the transformative interpretation of AI-mediated education remains contested rather than settled. Critical educational technology scholarship warns that periods of rapid technology adoption often generate solutionist narratives in which efficiency, scale, and innovation are treated as self-evident educational goods, whilst questions of labour, institutional control, public accountability, and unequal access receive less attention (Williamson et al., 2020). The 2023 Global Education Monitoring Report similarly emphasises that educational technology debates are divided and that technology can be beneficial, neutral, or harmful depending on governance, teacher preparation, access, and pedagogical purpose (Global Education Monitoring Report Team, 2023). The present review therefore treats educational transformation as an empirical and normative question, not as an assumed consequence of AI adoption.

The questions raised by artificial intelligence are not entirely new to educational theory. Long before the emergence of contemporary AI systems, educational scholars debated how knowledge is produced, mediated, and legitimised within social institutions (Javadinejad and Davari, 2026). Dewey conceptualised learning as an adaptive process grounded in experience, inquiry, and participation in democratic communities (English, 2023). Vygotsky emphasised the socially mediated nature of cognition and the role of cultural tools in extending human learning and development (Rigopouli et al., 2025). Freire challenged hierarchical models of knowledge transmission and argued for dialogical forms of education that position learners as active participants in knowledge creation rather than passive recipients of information (Harris and Roter, 2024). Similarly, Bourdieu’s analysis of cultural reproduction and Foucault’s examination of power–knowledge relations provide important perspectives for understanding how algorithmic systems may shape educational opportunity, access to knowledge, and institutional authority (Christensen, 2024). More recently, socio-constructivist and connectivist traditions have highlighted learning as a distributed process emerging through interactions amongst individuals, communities, technologies, and information networks (Lu et al., 2025). These theoretical perspectives collectively suggest that educational transformation is not simply a technological phenomenon but also an epistemological, social, cultural, and political process.

The proposed framework is therefore positioned as an integrative extension of existing educational theories, not as a replacement for them. Deweyan inquiry explains learning as connected to experience, uncertainty, and democratic problem-solving; Vygotskian mediation explains cognition as extended through tools and social interaction; Freirean critical pedagogy explains learners’ dialogic agency in knowledge production; socio-constructivist and connectivist traditions explain learning as emerging through networks of people, technologies, and information flows; nonlinear pedagogy explains adaptive rather than strictly sequential learning pathways; and critical data and AI literacy scholarship explains learner and educator agency over datafied learning environments (Atenas et al., 2025). The distinctive contribution of adaptive epistemic ecosystems lies in bringing these traditions into a single AI-sensitive frame that explicitly links pedagogical adaptation, algorithmic mediation, epistemic validation, governance, and institutional transformation. In contrast to models that focus primarily on individual cognition, networked learning, or nonlinear task design, the framework asks how educational knowledge is produced, authorised, contested, and governed when intelligent systems participate in assessment, curriculum recommendation, feedback, and institutional decision-making.

Despite increasing scholarly attention to AI in education, much of the existing literature remains fragmented. Reviews frequently focus on specific applications such as intelligent tutoring systems, learning analytics, generative AI, automated assessment, or student support technologies. Whilst these studies provide valuable insights, they often examine technologies in isolation and primarily evaluate effectiveness, efficiency, engagement, or adoption outcomes (Kuchynska et al., 2025; Mengliyev et al., 2024; Yue et al., 2022). Less attention has been devoted to understanding how these developments collectively influence the broader organisation of educational systems, including curriculum design, pedagogical authority, institutional governance, and the processes through which knowledge is validated and circulated. The unresolved problem is therefore not the complete absence of integrative models, but the limited specification of how AI-mediated adaptation, learning-data infrastructures, human-AI role allocation, epistemic validation, institutional governance, and justice interact within a single empirically examinable analytical scheme.

This limitation is particularly significant because AI-mediated educational transformation unfolds within highly unequal global contexts. Much of the existing evidence originates from technologically advanced educational systems in North America, Europe, East Asia, and other well-resourced settings (Toha, 2026). Consequently, important perspectives from the Global South, indigenous educational traditions, multilingual learning environments, and decolonial scholarship remain comparatively underrepresented (Galante et al., 2024). These perspectives are essential because educational technologies do not operate independently of social, cultural, linguistic, and historical conditions. Questions concerning whose knowledge is represented, whose values are embedded within algorithmic systems, and who benefits from technological innovation are central to understanding the broader educational implications of AI. Greater attention to epistemic diversity is therefore necessary if future educational systems are to avoid reproducing existing inequalities through new technological mechanisms.

This imbalance also affects the reference landscape of AI-in-education studies. Earlier systematic mapping of AI applications in higher education found that educational questions and educator perspectives were often less visible than technical design and performance evaluation (Zawacki-Richter et al., 2019). Political-economy analyses further show that AI education systems are shaped by state strategy, platform markets, regional policy networks, and private-sector infrastructures, rather than by pedagogy alone (Knox, 2020). Decolonial AI-ethics scholarship adds that globally circulating AI governance frameworks may reproduce Eurocentric assumptions when they are treated as universal templates (Nemorin, 2026). These critiques informed the citation audit and the final interpretation of the review, especially where the available evidence was technologically positive, English-language dominant, or conceptually aligned with the authors’ proposed framework.

Similarly, the ethical implications of AI in education extend beyond concerns about privacy, transparency, or technical bias (Mishara, 2024). Contemporary debates in critical AI ethics, ethics of care, responsible innovation, capability approaches, and decolonial ethics evaluate educational technologies according to their effects on human flourishing, agency, inclusion, cognitive autonomy, and social justice. These perspectives suggest that the central challenge is not simply whether AI systems function effectively, but whether they contribute to educational environments that expand learners’ capabilities, support diverse forms of knowledge, and promote equitable participation in knowledge creation (Akgun and Greenhow, 2022). Such considerations inform the review’s treatment of governance, ethics, and epistemic justice as constitutive rather than peripheral dimensions of AI-mediated educational change.

This review addresses these gaps through five interrelated domains: AI-enabled pedagogy and adaptive learning, curriculum transformation, learning analytics and assessment, human-AI collaboration and teacher authority, and governance, ethics, and epistemic justice. It does not treat adaptive epistemic ecosystems or post-linear pedagogy as predefined inclusion categories. Instead, it asks whether recurring relationships across the reviewed evidence require an integrative conceptual account. The labels adaptive epistemic ecosystem and post-linear pedagogy are reserved for the synthesis developed after inductive theme generation and are subsequently operationalised only as a second-pass interpretive test. Specifically, the review aims to: (1) examine how recent scholarship conceptualises the transformative role of artificial intelligence in education; (2) synthesise evidence from January 2020 to January 2026 on AI-associated changes in pedagogy, curriculum design, learning analytics, assessment, and institutional governance; (3) identify recurring cross-domain relationships amongst adaptive feedback, curriculum sequencing, assessment, human-AI roles, knowledge validation, and accountability; and (4) develop an evidence-bounded conceptual interpretation of these relationships whilst distinguishing current evidence from prospective research propositions.

## 2 Methodology

### 2.1 Review design and research questions

This study employed a systematic narrative review design to examine how artificial intelligence is reshaping educational systems, pedagogical processes, knowledge production, and institutional governance. A systematic narrative design was selected because the literature includes empirical investigations, conceptual papers, design-based studies, policy analyses, and prior reviews that cannot be aggregated meaningfully through meta-analysis. The review was guided by four questions: How has recent scholarship conceptualised the transformative role of artificial intelligence in education? What evidence exists regarding AI-associated changes in pedagogy, curriculum design, learning analytics, assessment, and institutional governance? What recurring relationships are reported amongst adaptive feedback, curriculum sequencing, assessment, human-AI roles, knowledge validation, and institutional accountability? What ethical, epistemological, and governance challenges accompany increasing human-AI participation in educational systems? These questions defined the substantive scope of the search and extraction process without prespecifying adaptive epistemic ecosystems or post-linear pedagogy as inclusion criteria or first-pass coding categories.

### 2.2 Review protocol and information sources

The review process followed PRISMA 2020 principles to improve methodological transparency, reproducibility, and auditability. Before screening, the review team defined the objectives, databases, time frame, eligibility criteria, screening stages, appraisal criteria, and synthesis approach. Searches were conducted across Scopus, Web of Science Core Collection, ERIC, ScienceDirect, and Google Scholar because these sources provide broad coverage of educational research, educational technology, artificial intelligence, learning sciences, public policy, and interdisciplinary scholarship. Database searching was complemented by backward and forward citation tracking of highly relevant papers to identify additional studies that may not have been captured through keyword searches alone. The search covered literature published between January 2020 and January 2026. Foundational theoretical sources published before 2020 were retained only for conceptual framing, including classical educational theory, and were not counted as part of the 124-study review corpus.

### 2.3 Search strategy

The search strategy was developed through preliminary scoping searches and refinement of terms related to artificial intelligence, educational processes, and systemic change. The generic Boolean structure was: (“artificial intelligence” OR AI OR “generative AI” OR “machine learning” OR “intelligent tutoring system*” OR “learning analytics” OR “adaptive learning” OR “educational data mining”) AND (education OR learning OR teaching OR pedagogy OR curriculum OR assessment OR “higher education” OR “K-12”) AND (transformation OR governance OR ethics OR personalisation OR personalization OR “human-AI collaboration” OR ecosystem OR “teacher agency” OR “algorithmic accountability”). Supplementary searches deliberately included critical and equity-oriented terms such as algorithmic bias, surveillance, data colonialism, epistemic injustice, teacher autonomy, digital divide, Global South, multilingual education, decolonial AI, explainability, accountability, and platform governance. Database-specific adaptations were made to titles, abstracts, and keywords where available. The labels adaptive epistemic ecosystem and post-linear pedagogy were not mandatory search terms and did not determine eligibility. Nevertheless, several transformation-block terms overlap semantically with the later conceptual vocabulary. This potential construct-alignment bias is therefore acknowledged explicitly: the review does not claim that all final themes emerged from a concept-free search, and framework-level conclusions are treated as interpretive syntheses rather than as purely inductive discoveries. Google Scholar was used only as a supplementary sensitivity source and was not treated as equivalent to the bibliographic database core. The retained review archive does not preserve a fixed rank cut-off, immutable result snapshot, or date-stamped ranking output for the Google Scholar component; exact rank-by-rank replication is therefore not claimed. Reproducibility claims are restricted to Scopus, Web of Science Core Collection, ERIC, and ScienceDirect, whilst Google Scholar and backward and forward citation tracking are reported as supplementary discovery procedures. No prevalence estimate, evidence-density count, or claim of thematic dominance is derived from Google Scholar rank order. Searches were limited to English-language publications, and that restriction is treated as a substantive limitation rather than evidence of global representativeness. Records were exported to a reference management file, duplicates were removed, and the resulting records were screened against the eligibility criteria.

### 2.4 Eligibility criteria

Studies were included if they met all of the following criteria: they addressed the application, implication, governance, ethics, or conceptualisation of artificial intelligence within formal, informal, or professional educational contexts; they were published from January 2020 through January 2026; they appeared in peer-reviewed journals, scholarly conference proceedings, academic books, book chapters, or authoritative policy reports; and they contributed empirical, theoretical, conceptual, methodological, or review-based evidence relevant to the research questions. Studies were excluded if they focused exclusively on technical algorithm development without educational application, were unrelated to learning or educational governance, were editorials, news articles, opinion pieces, blog posts, or non-scholarly commentary, lacked sufficient methodological or conceptual detail, duplicated another included record, or made claims about AI in education without an identifiable evidential basis. Earlier theoretical works were used selectively to situate concepts such as social learning, inquiry, power-knowledge relations, and educational agency, but these sources were not treated as part of the contemporary evidence sample.

### 2.5 Study selection process

**Figure 1:**
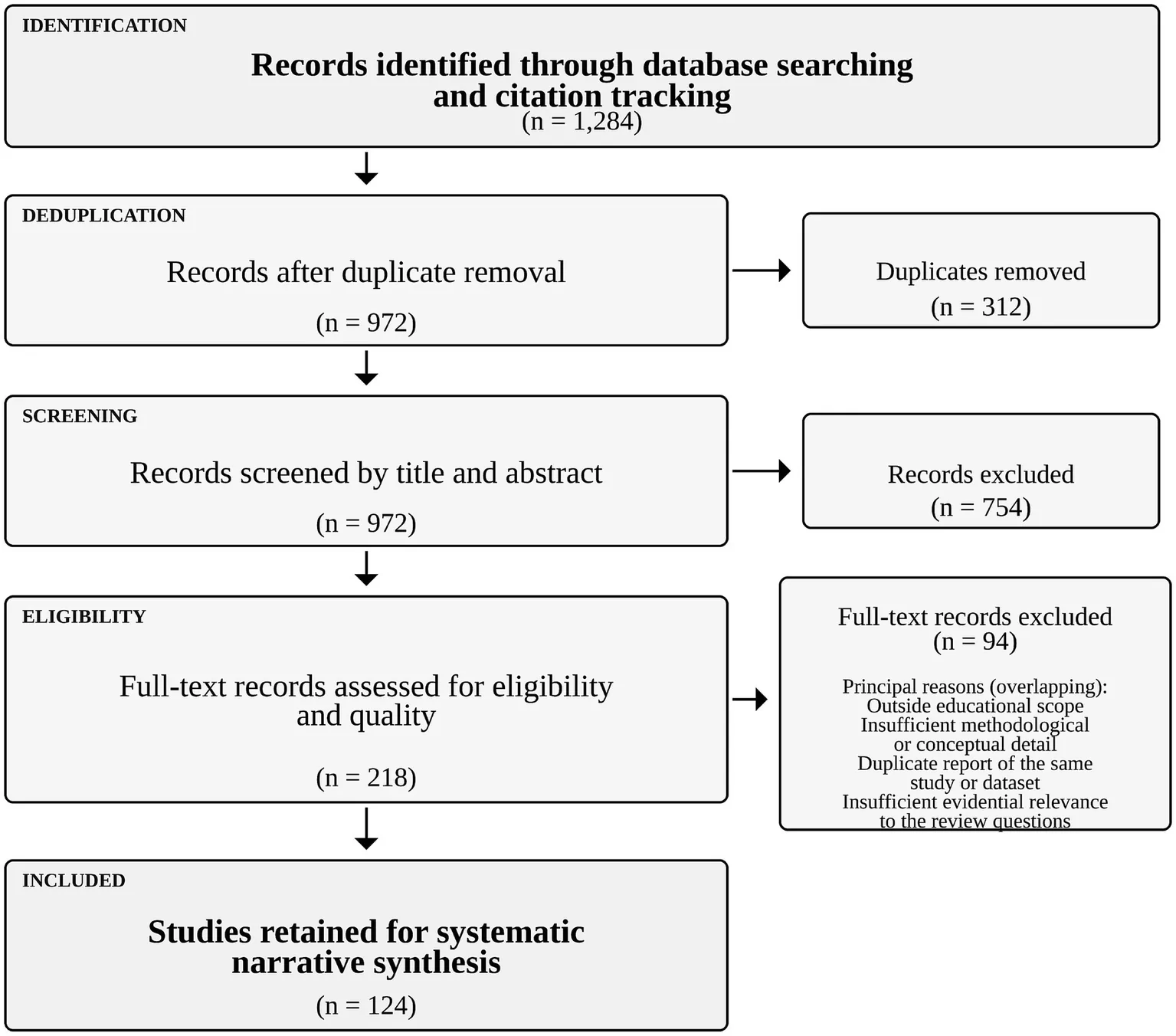
PRISMA-informed flow of study identification, screening, full-text assessment, and inclusion. Full-text exclusion reasons overlap; reason-specific counts were not preserved in the review record.

Study selection proceeded in four documented stages. First, records identified through database searching and citation tracking were imported into a reference management system and exact duplicates were removed. The initial search identified 1,284 records; 312 duplicates were removed, leaving 972 records for title and abstract screening. Second, 754 records were excluded because they did not meet the predefined topical or evidential eligibility criteria, leaving 218 records for full-text assessment. Ambiguous title-and-abstract records were retained for full-text assessment rather than excluded prematurely. Third, the 218 full-text records were assessed against the eligibility and type-appropriate quality criteria. Ninety-four records were excluded for one or more reasons, including being outside the educational scope, insufficient methodological or conceptual detail, duplicate reporting of the same underlying study or dataset, or insufficient evidential relevance to the review questions. These reasons were not logged as mutually exclusive numerical categories, so a reason-specific breakdown cannot be reconstructed reliably and is not inferred retrospectively. Fourth, 124 studies were retained for synthesis. Selection and appraisal were conducted collaboratively by the author team under shared criteria rather than through a formally documented dual-independent screening protocol; uncertain full-text decisions were discussed until consensus was reached. The review archive does not preserve stage-by-stage author assignments or duplicate-independent screening records for every item, so individual screening decisions cannot be attributed retrospectively to named reviewers and an inter-rater coefficient cannot be calculated defensibly. Figure 1 summarises the identification, screening, full-text assessment, and inclusion process. With the additional comparator literature used for theoretical positioning, the reference list contains 160 unique sources: 124 sources in the formal synthesis corpus and 36 methodological, foundational, theoretical, policy, contextual, or framework-comparison sources.

### 2.6 Quality appraisal

Quality appraisal was undertaken before synthesis using criteria appropriate to source type. Empirical studies were considered in relation to clarity of objectives, fitness of the design for the research question, transparency of sampling and data collection, traceability of analysis, and whether the interpretation was supported by the reported evidence. Review papers were assessed for search transparency, eligibility criteria, synthesis procedure, and alignment between evidence and conclusions. Conceptual and theoretical papers were assessed for definitional clarity, argumentative coherence, engagement with relevant literature, and the evidential basis of their claims. Policy reports were considered in relation to institutional authority, evidential grounding, transparency of assumptions, and relevance to educational practice or governance. Appraisal was conducted within the same collaborative author-team process used for full-text selection; it was not recorded as a duplicate-independent appraisal exercise. In this review, a substantial methodological or conceptual weakness means a limitation severe enough to prevent a source from supporting the type of claim for which it would otherwise be used, rather than the presence of any minor reporting imperfection. All 124 retained sources met the minimum type-appropriate threshold; no high/medium/low quality categories, pooled score, or numerical study weight was assigned. Quality influenced synthesis through claim calibration: empirical findings, review-level evidence, conceptual argument, design propositions, and policy analysis were not treated as equivalent forms of support. Because the full-text exclusion log recorded overlapping reasons rather than mutually exclusive quality-only categories, a separate number of studies excluded solely for quality cannot be reported defensibly. Journal indexing in Scopus or Web of Science was not used as a proxy for methodological quality; database indexing supported discovery, whilst admissible conference papers, books, book chapters, and authoritative reports were appraised according to their own evidence standards.

### 2.7 Data extraction

Data extraction was conducted using a structured review matrix to enable systematic comparison across heterogeneous sources. The matrix recorded bibliographic details, year of publication, geographical context, educational level, study type, AI application or construct, data source or evidence type, methodological approach, principal findings, reported opportunities, reported risks, and implications for pedagogy, assessment, curriculum design, governance, teacher agency, learner agency, and knowledge production. Each extracted item was linked to one or more review questions. This approach allowed conceptual and empirical studies to be compared without treating all forms of evidence as equivalent. It also enabled the review to distinguish between directly reported findings, author interpretation, and the present review’s synthesis-derived framework. Primary evidence function was also coded for corpus description as empirical, review/synthesis, conceptual/theoretical, design/methodological, or policy/report evidence. Educational sector, explicit geographical setting, methodological approach, AI/application type, and disciplinary context were recorded where they were identifiable from the study itself; categories that were not applicable to conceptual or cross-context sources were retained as not geographically or sectorally bounded rather than forced into a single location or discipline.

Following the initial inductive extraction and development of higher-order themes, the two central constructs were operationalised in a second interpretive pass to test whether the emerging conceptual labels were supported by the evidence rather than imposed on it. Evidence was mapped as relevant to adaptive epistemic ecosystems only when it addressed at least two of the following dimensions: adaptive feedback between learners and systems; explicit human-AI role negotiation; data infrastructures that shaped pedagogical or institutional decisions; mechanisms for epistemic validation; governance or accountability arrangements; and documented effects on curriculum, assessment, teacher agency, or learner agency. Evidence was mapped as relevant to post-linear pedagogy only when it described non-sequential learning pathways, learner-specific progression, iterative feedback cycles, emergent curriculum sequencing, or assessment embedded within learning rather than positioned only at the end of instruction. Studies that merely described digital delivery, online learning, or generic AI adoption were not treated as evidence for either construct unless they demonstrated these operational features. This second-pass operationalisation separated synthesis-derived interpretation from ordinary technology integration and made the proposed relationships available for subsequent empirical testing.

### 2.8 Data synthesis

A thematic narrative synthesis was undertaken because the evidence base was methodologically diverse and conceptually heterogeneous. The synthesis followed four steps. First, included studies were read repeatedly to identify recurring findings, constructs, tensions, and counterclaims. Second, extracted evidence was coded inductively according to the educational process addressed, the role assigned to AI, the level of educational organisation, and the reported risks or opportunities. Third, related codes were grouped into higher-order themes. Fourth, the themes were compared against the review questions to assess whether they supported, qualified, or contradicted the emerging interpretation of AI-mediated educational transformation. To reduce conceptual overlap, themes were retained only when they performed a distinct analytical function. Five interconnected but non-identical thematic domains emerged: AI-enabled curriculum transformation, adaptive and personalised learning pathways, continuous and data-driven assessment, reconfiguration of pedagogical authority and human-AI collaboration, and governance, ethics, and algorithmic epistemology. The labels adaptive epistemic ecosystem and post-linear pedagogy were articulated only after this inductive theme development and were then subjected to the second-pass operational test described above. The framework is therefore synthesis-derived rather than an *a priori* template guiding inclusion or initial coding. It is presented as an interpretive integration of recurring patterns, qualifications, and counter-evidence, not as a claim that all education systems have already reached this stage.

### 2.9 Evidence mapping, citation audit, and claim calibration

To address source-selection, citation-accuracy, and interpretive-bias concerns, the synthesis included an evidence-mapping and citation-audit step. Each major claim was checked against the extracted evidence to determine whether it was supported by empirical findings, conceptual argument, policy analysis, or prior review evidence. Claims supported mainly by conceptual literature were phrased as interpretive propositions, whereas claims supported by empirical studies were phrased as evidence-based patterns. Citations were reviewed for topical alignment with the statement they supported, and studies outside the stated contemporary review period were retained only when they provided foundational theoretical context. A second pass specifically examined whether the manuscript overrepresented AI-positive, English-language, recent, or conceptually aligned sources. This pass led to stronger incorporation of cautionary, critical, political-economy, decolonial, and policy-oriented scholarship, including sources that question whether AI adoption necessarily produces educational improvement (Nemorin, 2026; Williamson et al., 2020). This step prevented the conclusions from extending beyond the evidence presented and maintained a clear distinction between the reviewed literature and the authors’ conceptual contribution.

The corpus-level audit provides a descriptive account of the 124 sources retained for synthesis. The mapped corpus contains 8 sources from 2020, 7 from 2021, 8 from 2022, 16 from 2023, 32 from 2024, and 53 from 2025. By publication format, it comprises 108 journal articles, 9 conference papers, 4 book chapters, 2 books, and 1 other scholarly article. Primary evidence-function coding identified 46 empirical studies, 14 review or synthesis papers, 49 conceptual or theoretical contributions, 14 design, system-development, or methodological papers, and 1 policy report. A conservative primary-sector recoding assigned a single educational setting to 41 sources: 23 higher-education studies, 11 K-12 or primary-secondary studies, 4 teacher-education or professional-development studies, and 3 professional, vocational, or adult-learning studies; the remaining 83 sources were cross-sector, review/conceptual, or not bounded to one sector without inference. A geographically bounded setting could be assigned without inference to 17 sources; these included Asian (11), North American (4), African (2), Latin American (2), and Oceanian (1) settings, with three sources spanning more than one regional category. Application-level coding was non-mutually exclusive: 40 sources addressed adaptive learning, intelligent tutoring, or personalisation; 20 addressed learning analytics, prediction, or data-driven modelling; 11 addressed generative AI, large language models, or AI agents; 37 addressed assessment or feedback; 9 addressed human-AI collaboration or hybrid intelligence; 48 addressed governance, ethics, equity, or data justice; and 42 addressed curriculum or pedagogical design. Primary disciplinary focus was coded conservatively as mathematics, STEM, or physical-science education (16), language or literacy (10), teacher education or pedagogy (7), professional, vocational, or management education (5), arts, design, or creativity (3), medical or health education (1), and general or cross-disciplinary education (82). These descriptive categories characterise corpus composition rather than effect size or global representativeness; application categories are intentionally non-mutually exclusive, whilst sector, geography, and discipline were left unassigned where the source did not support a specific classification. Four 2026 publications cited in the synthesis sections postdate the January 2026 search cutoff and are treated as contextual updates rather than members of the 124-source synthesis corpus; pre-2020 sources and comparator literature used solely for theoretical positioning, including the sociotechnical and hybrid-intelligence comparators, are likewise excluded from corpus-frequency calculations. Evidence density was mapped across six manuscript-level synthesis domains: post-linear curriculum and emergent knowledge processes were supported by 46 mapped sources, systems and adaptive epistemic ecosystem arguments by 39, human-AI cognition by 17, ethical and pedagogical risks by 14, global trajectories by 8, and institutional authority by 6. These counts are thematic rather than mutually exclusive and indicate relative evidence density, not effect size, methodological quality, or global prevalence.

### 2.10 Limitations of the review

Several limitations shape the interpretation of this review. Restricting the search to English-language publications may have excluded relevant scholarship from national journals, regional repositories, multilingual education debates, and non-English policy literatures. This limitation is especially consequential for claims concerning the Global South, multilingualism, indigenous knowledge, decoloniality, and epistemic justice. The evidence synthesised here represents English-accessible scholarship about these contexts, not a comprehensive account of scholarship produced within them. Low representation of a region, language, or epistemic tradition in the corpus therefore cannot be interpreted as evidence that relevant scholarship or practice is absent. Cross-regional claims are consequently framed as patterns visible within the accessible evidence rather than as estimates of global prevalence. The evidence base also contains publication and optimism bias: successful pilots, platform evaluations, design proposals, and conceptually affirmative papers are more visible than failed implementations, abandoned systems, teacher resistance, or negative long-term outcomes. Many empirical studies assess short-term engagement, satisfaction, accuracy, or performance indicators rather than durable changes in learning, agency, equity, or institutional governance. Conceptual and policy papers add breadth, but they cannot substitute for longitudinal, comparative, and context-sensitive empirical evidence. Google Scholar contributed useful sensitivity but its dynamic ranking limits exact rank-by-rank reproducibility. The inclusion of empirical, conceptual, review-based, and policy-oriented literature also introduced heterogeneity that limited direct comparison and made meta-analysis inappropriate. The review therefore does not provide a pooled estimate of AI effectiveness, a universal model of educational transformation, or a deterministic prediction of future schooling. Its contribution is a transparent and critically bounded synthesis that identifies plausible patterns, counter-evidence, unresolved debates, and boundary conditions requiring further empirical testing. Methodological reproducibility is also limited by two features of the retained review record: stage-specific reviewer assignment and inter-rater statistics were not preserved, and Google Scholar did not have a fixed rank cut-off or immutable result snapshot. These omissions are not filled by retrospective assumptions; they define the boundary of what can be reproduced exactly from the review archive.

## 3 Evidence synthesis I: system-based, data-driven, and ecosystem-oriented accounts

The breadth of the review is integrative rather than encyclopaedic. The corpus was designed to connect evidence across pedagogy, curriculum, assessment, human-AI roles, institutional governance, and epistemic justice, but it does not provide equal representation of every educational level, region, technology, or research design. The corpus-level mapping reported above demonstrates this uneven evidence density. Accordingly, claims in comparatively lower-density domains are treated as emerging or provisional patterns rather than dominant or universally consistent findings. This calibration narrows the interpretive reach of the framework whilst retaining the cross-domain scope needed to examine systemic educational change.

**Figure 2:**
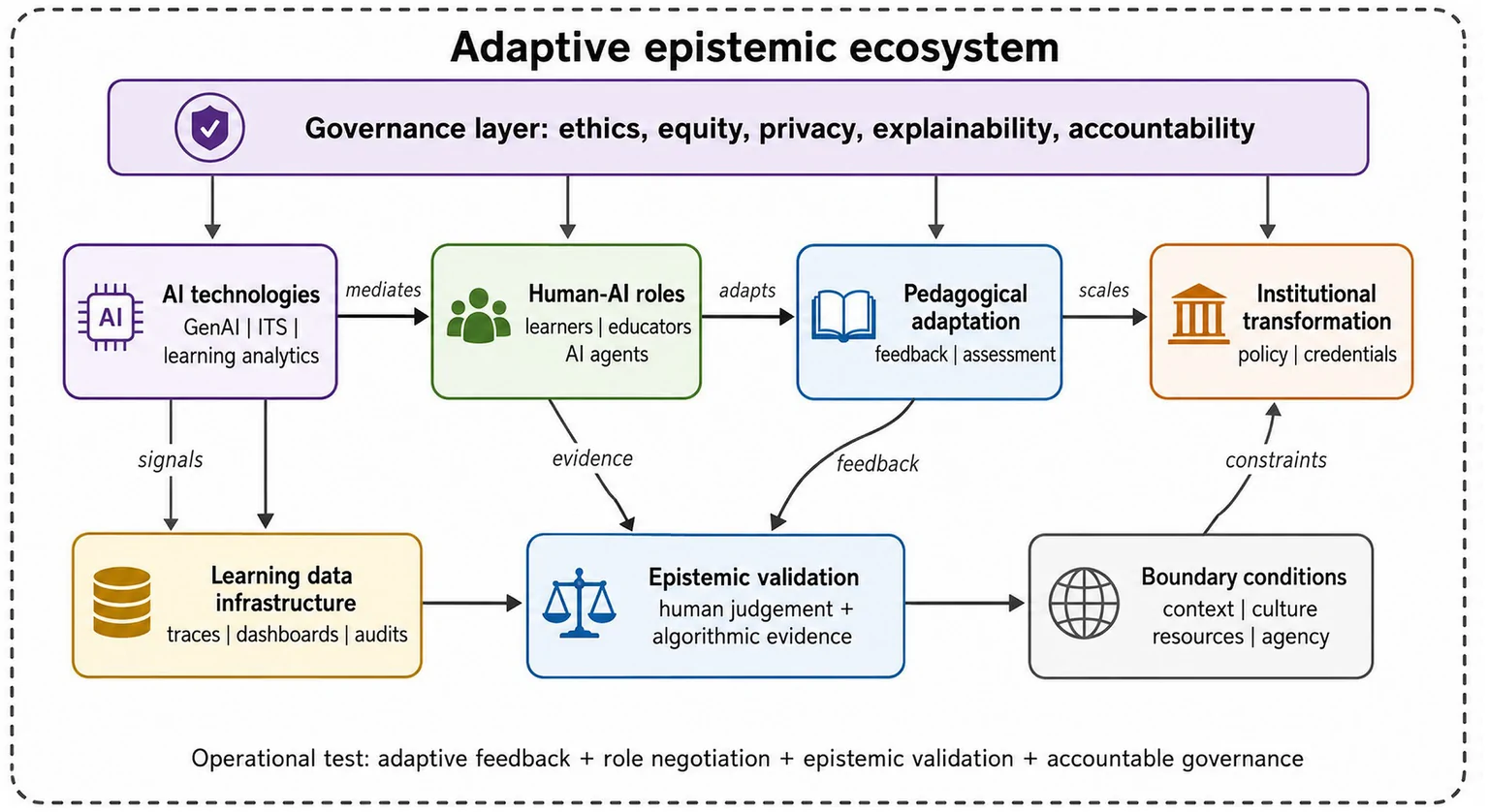
Conceptual framework linking AI technologies, governance, pedagogical adaptation, and institutional transformation.

The reviewed corpus contains both tool-centred AI education models and broader accounts of adaptive, data-mediated educational ecosystems. The evidence does not support a simple claim that all educational systems are already transformed. Rather, it shows an uneven distribution of approaches: many AI applications remain embedded in system-based models of optimisation, whilst learning analytics, adaptive platforms, teacher-agency studies, and governance debates increasingly point towards more relational and ecosystem-oriented forms of educational organisation. Figure 2 operationalises this synthesis by linking AI technologies, learning data infrastructures, human-AI roles, pedagogical adaptation, governance, epistemic validation, and institutional transformation. The diagram rejects technological determinism by placing governance and boundary conditions around AI-enabled adaptation. It identifies the relationships that require empirical examination if AI-mediated education is to be understood as an adaptive epistemic ecosystem rather than as isolated platform adoption.

### 3.1 Limits of system-based AI education models

The framework is distinguished from adjacent models at the outset. Conventional systems theory is useful for describing instructional inputs, processes, outputs, and feedback, but it often assumes bounded control and predefined performance criteria. Socio-constructivism explains the social production of knowledge, but it does not by itself account for algorithmic prediction, automated assessment, or data-driven governance (Tran et al., 2025). Connectivism explains distributed learning across networks, but it gives less attention to institutional accountability and epistemic justice (Corbett and Spinello, 2020). Nonlinear pedagogy explains emergent learning trajectories, but it does not necessarily specify how AI infrastructures, data models, and governance regimes shape those trajectories. Learning analytics frameworks explain data-informed feedback and prediction, yet they can remain focused on measurement rather than knowledge legitimacy. The adaptive epistemic ecosystem framework contributes a more integrated account by treating AI technologies, human actors, epistemic validation, institutional policy, and justice-oriented governance as co-constitutive elements of educational change.

A more direct comparison with adjacent integrative frameworks further narrows the contribution. Educational ecosystem approaches emphasise nested interdependence amongst learners, institutions, communities, and contexts. In higher education, an AI Ecological Education Policy Framework already integrates pedagogical, governance, and operational dimensions (Chan, 2023). Sociotechnical approaches emphasise that AI cannot be separated from human practises, institutional arrangements, infrastructures, and collective imaginaries, and recent work in education explicitly develops this argument for AI integration (Mamlok, 2025). Postdigital perspectives reject a simple digital versus non-digital divide and treat educational practice as materially and technologically entangled; learning-analytics frameworks foreground data capture, modelling, prediction, and feedback. Hybrid-intelligence perspectives likewise predate the present framework: hybrid human-AI regulation has been proposed specifically to support self-regulated learning in adaptive educational technologies (Molenaar, 2022). Critical-AI frameworks foreground power, bias, accountability, labour, and justice. A multidisciplinary AI-and-education agenda further combines educational ecology, socio-technical assemblage, postdigital conditions, and micro-meso-macro levels, demonstrating that ecosystem thinking itself is not novel (Jaldemark et al., 2025). The adaptive epistemic ecosystem framework therefore does not claim to originate ecosystem thinking, socio-technical analysis, postdigital entanglement, hybrid intelligence, or critical governance. Its narrower contribution is to connect these strands through an explicit set of relationships amongst adaptive feedback, learning-data infrastructure, human-AI role negotiation, curriculum and assessment, epistemic validation, governance, and institutional boundary conditions, and to expose those relationships to empirical confirmation, qualification, or rejection.

The novelty claimed here is therefore integrative rather than elemental. Adaptive and personalised learning frameworks primarily explain how instructional content or feedback can be adjusted to learner needs; connectivist and socio-constructivist traditions explain networked and socially mediated knowledge formation; learning-analytics frameworks explain data capture, modelling, prediction, and feedback; nonlinear pedagogy explains flexible and emergent learning trajectories; and critical AI scholarship foregrounds power, bias, accountability, and justice. Adaptive epistemic ecosystems bring these usually separated concerns into one relational model by specifying how AI mediation, learning-data infrastructure, human-AI role allocation, pedagogical adaptation, assessment, epistemic validation, governance, and institutional scaling constrain and reshape one another. The framework is not proposed as a new adaptive algorithm, a replacement learning theory, or evidence that a new educational system already exists. Its contribution is a testable conceptual architecture for analysing the couplings amongst these components when AI participates in educational knowledge production and decision-making.

Historically, artificial intelligence in education (AIED) has been largely conceptualised through an engineering-oriented systems paradigm. Within this perspective, education is treated as a relatively bounded and controllable process composed of predefined inputs (curriculum content), processes (instructional delivery), and outputs (learning outcomes). AI technologies are consequently deployed as subsystems such as intelligent tutoring systems, adaptive learning platforms, or automated assessment tools designed to optimise instructional efficiency, personalise content delivery, and improve measurable learning performance. Empirical evidence indicates that these approaches can produce meaningful educational benefits in specific contexts. For example, the systematic review by Garzón et al. (2025) reports positive effects of AI-supported educational systems on learning outcomes, engagement, and personalised instruction through adaptive pacing, automated feedback, and task sequencing mechanisms.

Despite these benefits, a substantial body of educational theory suggests that the systems paradigm offers a partial account of learning. The assumption that knowledge can be efficiently delivered through increasingly sophisticated instructional systems contrasts with long-standing educational traditions that conceptualise learning as an active, socially situated, and context-dependent process. As previously noted, Dewey conceptualises education as inquiry grounded in experience and reflective engagement with real problems (Quay, 2019). Vygotsky emphasises the role of social interaction, cultural tools, and mediation in cognitive development (Chen, 2025). Freire critiques transmission-based education and argues for dialogical pedagogies in which learners participate actively in knowledge construction rather than receiving information passively (Nugraha et al., 2024). These perspectives suggest that educational processes cannot be reduced to optimisation of instructional inputs and outputs, since learning is shaped by interaction, meaning-making, and agency. From socio-constructivist and connectivist perspectives, learning emerges through distributed networks of people, tools, institutions, and informational environments (Corbett and Spinello, 2020). This implies that educational outcomes are not fully predictable or controllable through predefined instructional pathways. Whilst AI systems may support adaptation and personalisation, their underlying design logic often prioritises optimisation of individual performance against predefined benchmarks rather than supporting collective knowledge construction or contextual epistemic development.

Critical scholarship has raised additional concerns regarding system-based AI models. Risks identified in AI-in-education research include algorithmic bias, reduced transparency, data privacy challenges, and weakening of meaningful pedagogical relationships (Al-Zahrani, 2024). These issues align with broader debates in critical artificial intelligence ethics, responsible innovation, and capability approaches, which emphasise agency, autonomy, justice, and the expansion of human capabilities in educational contexts rather than narrow performance gains. Questions of power and inequality further complicate system-based models. Political-economy analysis of AI and education shows that AI systems are shaped by state strategy, platform markets, institutional priorities, and socio-technical relations rather than by pedagogy alone (Knox, 2020). This makes inequality a property of institutional and infrastructural arrangements as well as of technical design. Foucault’s analysis of surveillance and power relations provides a complementary lens for understanding how AI systems introduce new forms of monitoring and classification that reshape institutional authority and educational governance (Sahakyan et al., 2025). These dynamics are particularly significant in contexts where algorithmic decision-making influences assessment, tracking, and learner progression.

The limitations of system-based approaches are also evident in global and culturally diverse contexts. Scholars working within decolonial, indigenous, and Global South perspectives argue that AI systems often reflect assumptions derived from technologically and culturally dominant regions (Mbah and Jarvis, 2026). This can result in the marginalisation of local epistemologies, multilingual practice, and context-specific forms of knowledge. As a result, AI systems that appear effective in one educational context may not translate meaningfully across diverse educational environments. This raises concerns about epistemic inclusion, cultural responsiveness, and the global equity of AI-mediated education.

### 3.2 Emergence of data-driven learning environments

The expansion of digital learning technologies has produced large-scale educational datasets, including clickstream logs, discussion contributions, assessment records, collaboration traces, and engagement metrics. This has contributed to a shift from system-based instructional design towards data-driven learning environments in which continuous data collection, analysis, and feedback play a central role. In these environments, technology functions not only as a delivery mechanism but also as an infrastructure for observing, modelling, and responding to learning processes in real time. Learning analytics research reflects this shift. Wang and Liang (2025) describe data-driven educational systems that integrate multiple data sources to generate comprehensive representations of student learning and to support evidence-informed pedagogical decision-making. Compared with traditional assessment models, these systems enable continuous monitoring of learner progress and more immediate instructional adjustments.

However, data-driven environments introduce important epistemological and methodological concerns. As algorithmic systems increasingly mediate interpretation of educational data, they begin to influence what is recognised as meaningful evidence of learning. This development can be understood as algorithmic epistemology, defined as the increasing role of computational systems in shaping how educational knowledge is produced, validated, and operationalised. In this context, AI systems do not only represent learning processes but also actively participate in defining them. Transparency and interpretability remain central challenges. Farrow (2023) argues that AI systems in education need to be understandable to all stakeholders, including educators, learners, and institutional decision makers. This principle of Explicable AI in Education is essential for ensuring trust, accountability, and meaningful human oversight in data-intensive educational systems.

At the same time, data-driven environments risk reinforcing reductionist interpretations of learning if quantitative indicators become the dominant basis for evaluation. Whilst learning analytics can provide valuable insights, overreliance on measurable proxies may marginalise dimensions of education that are difficult to quantify, such as creativity, ethical reasoning, collaboration, and cultural understanding (Mirzaei et al., 2025). Ethical frameworks such as ethics of care and responsible innovation emphasise holistic human development rather than the reduction of learning to performance optimisation (Hummel, 2025). Capability approaches similarly assess educational value in terms of the freedoms and opportunities learners have to develop meaningful capabilities.

Concerns about equity and epistemic diversity are also central. Research grounded in decolonial and Global South perspectives highlights that educational data infrastructures are often shaped by linguistic, cultural, and institutional contexts that do not reflect global diversity (Guzmán-Valenzuela et al., 2025). This raises concerns that data-driven systems may reproduce epistemic asymmetries by privileging dominant knowledge systems whilst marginalising local, indigenous, and multilingual educational practice. These issues underscore the importance of designing AI systems that are responsive to plural epistemologies rather than narrowly optimised for dominant contexts. Although data-driven environments represent a significant evolution from system-based models, they frequently retain an underlying logic of optimisation and prediction (Wang et al., 2025b). In education-specific applications, academic performance prediction and student-management systems similarly prioritise prediction, monitoring, and targeted intervention (Pan et al., 2025; Wang, 2025). In this sense, data-driven environments remain analytically distinct from fully ecosystem-oriented accounts because expanded monitoring and prediction do not by themselves establish integrated governance, epistemic validation, or negotiated human-AI roles.

Critical data and AI literacy scholarship extends this concern by shifting attention from access to participation, agency, and data justice. Atenas et al. argue that learners and educators need opportunities to examine how data are produced, classified, interpreted, and mobilised within educational decision-making (Atenas et al., 2025). This perspective strengthens the present framework because an adaptive epistemic ecosystem cannot be evaluated only by the accuracy of its predictions. Its educational value also depends on whether participants understand, question, and reshape the data practice through which educational knowledge is made actionable.

System-based models focus primarily on instructional delivery, whilst data-driven environments emphasise monitoring, prediction, and feedback. In contrast, adaptive epistemic ecosystems are conceptualised as environments in which knowledge production, pedagogical practice, assessment, governance, and technological mediation co-evolve dynamically through interactions amongst human actors and AI systems (Punziano, 2025). This framework shifts attention from optimisation of individual components towards the relational and systemic dynamics of educational transformation. Table 1 summarises contemporary AI-enabled educational systems and platforms, including their subject domains, learner populations, algorithmic approaches, assessment strategies, and operational characteristics. Across the platform examples synthesised in Table 1, prediction, personalisation, monitoring, and performance optimisation recur more often than system-level governance or epistemic validation. This pattern supports a bounded comparison between optimisation-oriented applications and more integrative ecosystem-oriented accounts; it does not establish that all current AI applications belong to a single developmental stage.

**Table 1:** AI-education systems, platforms, and operational characteristics.

System/Model name	Learning area/Subject	Target learners	AI approach/Algorithm	Assessment type	Implementation level	Technology stack/Platform features	Data sources utilised	Governance/Control mechanism	Key publications/Studies
ALEKS	Mathematics	K-12, Higher Ed	Adaptive Learning, Knowledge Tracing	Formative, Competency-based	Classroom and Online	LMS integration, dashboards	Student responses, quizzes	Teacher-supervised	Fang et al. (2019); Kumor et al. (2024); Sun et al. (2021)
Knewton Adaptive Learning	STEM, Mathematics	K-12	Adaptive Learning, Predictive Analytics	Formative, Real-time	School-wide	Cloud platform, dashboards	Engagement metrics, assessment data	Hybrid	Akintola et al. (2025); Conklin (2016)
Squirrel AI Tutor	STEM, Mathematics	K-12	ITS, Knowledge Graph	Competency-based	School and District	Personalised dashboards, mobile support	Student knowledge maps, assessment logs	Centralised	Cui et al. (2018)
Carnegie Learning MATHia	Mathematics	K-12	ITS, Cognitive Modelling	Formative, Adaptive	Classroom	LMS, dashboards, predictive scoring	Student responses, logs	Teacher-supervised	Almoubayyed et al. (2023); Norberg et al. (2025)
Smart Sparrow (adaptive learning system, later integrated into Pearson)	STEM, Biology, Chemistry	Higher Ed	Adaptive e-learning, personalised feedback, mastery-based learning pathways	Formative, Continuous	University	eLearning platform, analytics	Quiz and activity data	Teacher-supervised	Linden et al. (2019); Mohammad Bagheri (2015)
Duolingo	Language Learning	K-12, Adult	NLP, Gamification, Reinforcement Learning	Continuous, Formative	Online Platform	Mobile apps, gamification, dashboards	Text/audio submissions, progress logs	Centralised	Ajisoko (2020); Ouyang et al. (2024); Shortt et al. (2023)
Century Tech	STEM, Literacy	K-12	Adaptive Learning, Data Analytics	Formative, AI-optimised	Classroom and School	LMS, dashboards, analytics	Engagement metrics, learning logs	Hybrid	Shashkova et al. (2025)
DreamBox Learning	Mathematics	K-8	ITS, Adaptive Learning	Formative, Real-time	Classroom	Cloud platform, dashboards	Clickstream, assessment logs	Teacher-supervised	Dutta et al. (2024); Foster (2024)
ASSISTments	Mathematics	Middle and High School	ITS, Immediate Feedback	Formative	Classroom	LMS, dashboards	Student response data	Teacher-supervised	Bazoukis et al. (2024); Feng et al. (2023)
Thinkster Math	Mathematics	K-12	Personalised Tutoring, AI Feedback	Formative	Online and School	Mobile app, dashboards	Student input, learning logs	Teacher-supervised	Alnaim and Al-Otaibi (2025)
Adaptive STEM Labs	STEM, INQUIRY-based Learning	K-12 and tertiary	AI-guided Simulation, Predictive Analytics	Formative	Classroom and Online	Virtual labs, dashboards	Interaction logs, simulation results	Teacher-supervised	Grammatikos et al. (2025); Kehinde (2023)
Lifelong Learning LMS	Professional Skills	Adults, career-focused	Adaptive Learning, Skill Gap Analysis	Competency-based, Formative	Regional/National	Cloud LMS, dashboards, micro-modules	Completion logs, skill assessments	Hybrid	Munna et al. (2024); Wea et al. (2023)

### 3.3 Synthesis-derived integration: adaptive epistemic ecosystems

The convergence of systemic limits and data-driven possibilities supports a conceptual transition towards theorising education as an adaptive epistemic ecosystem. In this review, the construct refers to an evolving network in which human learners, educators, AI agents, data repositories, assessment systems, and institutional structures interact recursively. Its operational features are adaptive feedback, distributed but accountable agency, transparent epistemic validation, and governance capable of constraining harmful forms of automation. The related concept of post-linear pedagogy refers to adaptive, iterative, and learner-specific learning pathways rather than a rejection of sequence, structure, or disciplinary progression. The concept therefore differs from simple personalisation: personalisation may optimise content for an individual, whilst post-linear pedagogy examines how curriculum, feedback, inquiry, peer interaction, and assessment co-evolve through multiple learning trajectories (Damaševičius, 2025). It also differs from generic connectivism by giving explicit attention to AI infrastructures, data governance, and institutional authority. Teachers are consequently conceptualised as epistemic designers who curate resources, interpret algorithmic evidence, protect learner agency, and sustain disciplinary standards, whilst AI systems function as bounded cognitive mediators rather than autonomous educational authorities (Chatzichristofis et al., 2025).

Within this review, post-linear pedagogy is not used as a synonym for adaptive learning, personalised learning, inquiry-based learning, or self-regulated learning. The distinction is analytical. Adaptive learning denotes system adjustment in response to learner performance or state; personalisation denotes tailoring of content, pace, or support to an individual; inquiry-based learning organises learning around questions, investigation, evidence, and explanation; and self-regulated learning concerns the learner’s planning, monitoring, evaluation, and regulation of learning. Post-linear pedagogy can incorporate each of these processes, but its distinctive object is the architecture of progression itself: curriculum, feedback, assessment, peer interaction, teacher judgement, and AI mediation can recursively alter the next learning step rather than merely optimise movement through a fully predetermined sequence. The concept therefore concerns how multiple educational processes co-evolve across a learning trajectory, whilst preserving disciplinary structure and accountable human judgement.

This integrative framing also generates empirically examinable relationships. First, adaptive feedback should support learning and agency more reliably when learners and educators retain meaningful role negotiation and the ability to contest algorithmic recommendations. Second, pistemic-validation practices should mediate the relationship between AI-generated recommendations and the knowledge claims ultimately accepted in teaching and assessment. Third, governance arrangements should moderate how data intensity affects privacy, trust, teacher agency, and equity. Fourth, institutional transformation should depend on boundary conditions such as infrastructure, professional capacity, culture, language, and policy rather than on AI availability alone. These propositions can be examined through longitudinal classroom studies, multilevel institutional analyses, comparative cross-context research, mixed-method designs, and algorithm or governance audits. The framework’s value is therefore open to empirical confirmation, qualification, or rejection.

**Figure 3:**
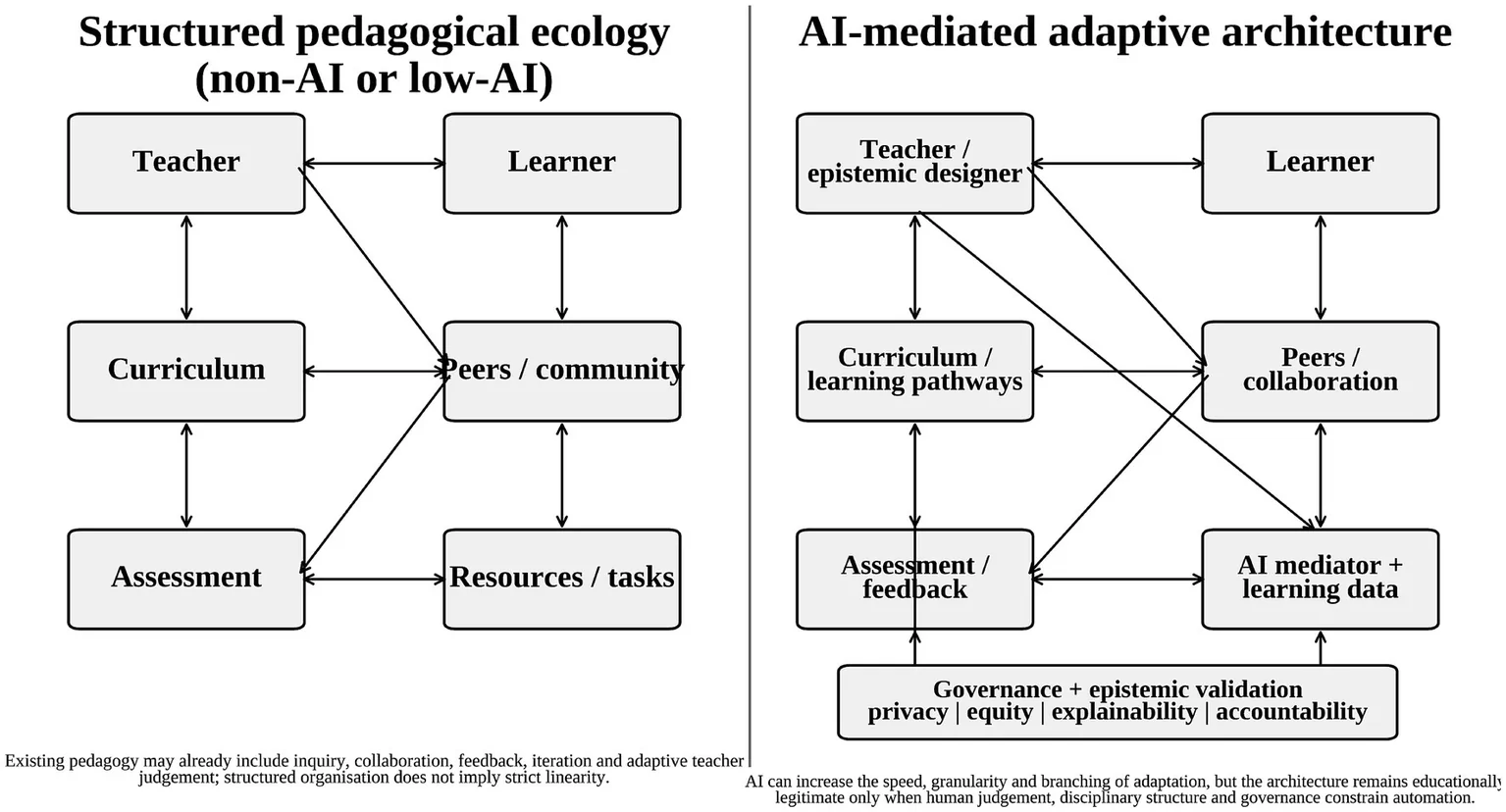
Comparison of structured pedagogical ecologies and AI-mediated adaptive learning architectures.

The ecosystem approach finds resonance in responses to complex educational challenges. Shah et al. (2024), arguing for a shift in education for displaced communities, explicitly advocate for an “ecosystems approach” based on “connectedness and nestedness,” “reflective learning,” and “working from potential rather than problems.” This mirrors the adaptive epistemic ecosystem’s emphasis on holistic connections, recursive feedback, and generative growth over deficit-based, linear solutions. The comparison therefore concerns differences in the organisation of feedback, agency, data, and governance rather than a historical progression from an allegedly linear form of conventional pedagogy to an inherently superior AI-mediated form (Figure 3).

## 4 Human-AI co-evolution and cognitive futures

Human-AI co-evolution, hybrid cognition, metacognitive regulation, and learner agency constitute the cognitive and relational dimension of AI-mediated education. This dimension is analytically distinct from the system-level discussion in Section 3 and the curriculum discussion in Section 5 because it focuses on how intelligence, judgement, reflection, and knowledge participation are distributed across human and artificial actors. The reviewed literature indicates movement from viewing AI as an instructional delivery tool towards understanding it as a cognitive mediator whose educational value depends on design, oversight, equity, and preservation of human judgement. Human-AI collaboration is therefore treated as an emerging and contested educational arrangement rather than as an automatically beneficial future.

### 4.1 Hybrid cognition and distributed intelligence

Hybrid intelligence refers to collaborative arrangements in which human and artificial agents contribute complementary cognitive capabilities to problem-solving, learning, and knowledge production. Within educational contexts, hybrid cognition can be understood as a form of distributed intelligence in which cognitive processes are shared across learners, educators, digital technologies, and institutional infrastructures. This perspective aligns with Vygotsky’s conception of cognition as mediated through cultural tools and social interaction, as well as connectivist perspectives that view learning as emerging through networks of human and technological actors rather than solely within individual minds.

Within the 17-source human-AI cognition domain, several conceptual and empirical contributions examine hybrid intelligence as a way of combining human contextual judgement, creativity, and ethical reasoning with computational speed, pattern recognition, and analytical capacity. The evidence is heterogeneous rather than uniformly convergent: some studies emphasise potential complementarity, whilst others identify coordination, trust, agency, and communication problems. Different architectural and conceptual approaches therefore require separate empirical evaluation.

Similarly, Liu and Fu (2024) argue that closer forms of collaboration between human and artificial intelligence are increasingly shaping creative and knowledge-intensive activities. Their integrative cognitive creation model suggests that interactions between human and machine intelligence may support new forms of iterative problem-solving, innovation, and knowledge generation. Rather than viewing AI as a replacement for human expertise, this perspective positions AI as a complementary participant in complex cognitive processes.

However, research grounded in distributed cognition theory demonstrates that effective hybrid intelligence cannot be reduced to technical architecture alone. Studies of human-AI collaboration reveal important social and cognitive challenges that complicate optimistic visions of seamless human-machine integration. Sidji et al. (2024), for example, examined human-AI cooperation in board-game environments and found that the presence of AI assistants could interfere with important human team processes, including shared mental model formation and theory-of-mind reasoning. These findings suggest that introducing AI into collaborative settings may alter communication patterns, trust relationships, and collective reasoning processes in ways that are not always beneficial.

From an educational perspective, these findings are significant because they indicate that successful hybrid cognition depends not only on computational performance but also on the social conditions that support learning and collaboration. Socio-constructivist theories emphasise that knowledge is co-constructed through interaction, dialogue, and participation (Lehtinen et al., 2023). Consequently, AI systems that disrupt these processes may undermine rather than enhance collective learning. The educational value of hybrid intelligence is therefore best understood as an emergent property of socio-technical interaction rather than as a direct consequence of technological sophistication alone.

Questions of equity and inclusion further complicate the development of hybrid intelligence. Critical AI ethics scholars have highlighted concerns regarding algorithmic bias, uneven access to AI resources, and the possibility that AI systems may privilege particular forms of knowledge and reasoning (Belenguer, 2022; Hanna et al., 2025). These concerns are particularly relevant in culturally and linguistically diverse educational settings where learners may engage with knowledge through different epistemic traditions. As a result, the effectiveness of hybrid intelligence is likely to depend not only on technical design but also on the extent to which systems accommodate diverse forms of participation, communication, and knowledge construction.

### 4.2 AI as a metacognitive regulator

A particularly significant development within hybrid cognition research is the reconceptualisation of AI from a provider of answers to a facilitator of metacognitive processes. Metacognition refers to the ability of learners to plan, monitor, evaluate, and regulate their own thinking and learning. Rather than encouraging cognitive outsourcing, this perspective positions AI as a mechanism for supporting reflective learning and self-regulation.

One of the most direct educational applications of this idea is presented by Tomisu et al. (2025) through their “Cognitive Mirror” framework. This model represents a shift from viewing AI as an authoritative source of information towards viewing it as a participant in the learning process. Using a Teaching Quality Index (TQI), the system modulates AI responses from deliberate uncertainty to scaffolded prompts, encouraging learners to explain concepts, identify gaps in understanding, and refine their reasoning. In doing so, the framework externalises aspects of learner thinking and transforms interactions with AI into opportunities for reflection and self-regulated learning. The educational significance of this approach lies in its emphasis on supporting learners’ metacognitive awareness rather than simply improving task performance.

This perspective resonates with Dewey’s conception of reflective inquiry, which emphasises the importance of deliberate reflection in learning, and with Vygotskian approaches that view scaffolding and guided participation as essential mechanisms for cognitive development. Rather than replacing human thought, AI systems operating as metacognitive supports may assist learners in becoming more aware of their own reasoning processes and learning strategies.

Related developments can be observed in broader AI research that seeks to embed metacognitive capabilities within AI systems themselves. Walker et al. (2025) argue that safe and responsible AI requires systems capable of monitoring, evaluating, and regulating their own cognitive processes. Their framework proposes mechanisms through which AI systems can assess confidence, identify potential errors, and adapt decision-making strategies. Similarly, the SOFAI architecture developed by Bergamaschi Ganapini et al. (2025) incorporates a metacognitive component that coordinates between rapid intuitive processing and slower deliberative reasoning processes, thereby improving decision quality and resource allocation. Although these studies focus primarily on AI autonomy and performance, they suggest a broader possibility in which educational environments consist of mutually adaptive participants. In such settings, learners engage in self-regulation whilst AI systems simultaneously monitor and adapt their own operations. This form of bidirectional metacognition has the potential to support more responsive and adaptive learning environments.

At the same time, important ethical considerations remain. Critical AI ethics and responsible innovation frameworks caution that systems designed to guide learner reflection may unintentionally encourage overreliance on algorithmic feedback or diminish opportunities for independent judgement (Yan et al., 2025). Ethics-of-care perspectives similarly emphasise educational relationships grounded in human support, empathy, and contextual understanding. Consequently, metacognitive AI is best viewed as a tool for supporting reflective learning rather than as a substitute for human autonomy, pedagogical expertise, or critical reasoning. These considerations suggest that the educational value of AI-mediated metacognition depends not only on technical functionality but also on careful pedagogical design. Effective systems enhance learners’ capacity for self-regulation whilst preserving their ability to question, challenge, and critically evaluate both human and algorithmic sources of guidance.

### 4.3 Learners as epistemic agents within intelligent environments

One of the most significant aspirations associated with human-AI collaboration in education is the strengthening of epistemic agency, defined as the learner’s capacity to participate in, evaluate, challenge, and contribute to processes of knowledge creation and validation. This conception aligns closely with Freire’s vision of learners as active co-creators of knowledge rather than passive recipients of information (Nwobodo, 2025). It also reflects broader socio-constructivist perspectives that position learning as participation in communities of inquiry and knowledge building.

Empirical research suggests that educational environments can be designed to support the development of epistemic agency. Nieminen et al. (2025), for example, examined a digitally mediated authentic assessment in a university archaeology course and found that students who created publicly accessible knowledge artefacts increasingly viewed themselves as contributors to knowledge rather than simply consumers of information. Similarly, Baze et al. (2025) demonstrated that learners engaged in design-based engineering projects were capable of negotiating roles, sharing epistemic authority, and taking ownership of ideas when working on authentic problem-solving tasks. These findings indicate that pedagogical design plays a critical role in shaping opportunities for learners to participate meaningfully in knowledge production.

However, the literature also demonstrates that epistemic agency is neither automatic nor evenly distributed. Baze et al. (2025) observed that changes in group composition could generate gendered power dynamics that constrained participation and limited some learners’ ability to contribute to collective reasoning processes. Such findings highlight that epistemic agency is socially negotiated and influenced by broader patterns of power, recognition, and inclusion. These observations resonate with Bourdieu’s analysis of cultural reproduction and with Foucault’s examination of power-knowledge relations. Both perspectives suggest that participation in knowledge production is shaped by social structures that privilege certain voices, forms of expertise, and ways of knowing over others. Consequently, educational technologies alone cannot guarantee equitable participation in learning (Bawa and Bawa, 2025). Without deliberate attention to inclusion and power relations, intelligent learning environments may reproduce existing inequalities even when they are designed to be learner-centred.

Questions of epistemic agency also extend beyond local classroom interactions. Scholars working within decolonial, indigenous, multilingual, and Global South traditions have argued that educational technologies frequently privilege dominant knowledge systems whilst marginalising local epistemologies and culturally situated forms of understanding (Omodan, 2025; Perales et al., 2026). From this perspective, meaningful participation in AI-mediated learning environments requires more than access to technological tools. It also requires recognition of diverse forms of knowledge and opportunities for learners to contribute from their own linguistic, cultural, and social contexts (Hossain, 2024). The capability approach reinforces this position by emphasising the expansion of individuals’ freedoms to develop and exercise meaningful forms of agency rather than merely improve measurable performance outcomes. These concerns highlight the importance of epistemic diversity within AI-mediated education. The goal is not simply to increase participation within existing knowledge structures but to create environments in which multiple forms of knowledge can be recognised, negotiated, and developed. Such an approach is particularly important in increasingly global educational ecosystems where learners engage across diverse cultural and epistemic traditions.

The literature suggests that hybrid cognition, metacognitive regulation, and epistemic agency represent complementary dimensions of emerging AI-mediated educational environments. Hybrid cognition concerns the distribution of cognitive activity across human and artificial actors. Metacognitive regulation concerns the monitoring and adaptation of learning processes (Xue and Khalid, 2026). Epistemic agency concerns learners’ capacity to participate meaningfully in the creation and evaluation of knowledge (Nieminen and Ketonen, 2024).

## 5 Post-linear curriculum and emergent knowledge

Curriculum and knowledge organisation form a separate analytical domain within the synthesis. Curriculum change is distinguished from the broader ecosystem argument in Section 3 and from the authority argument in Section 6 because it concerns how learning sequences, content boundaries, feedback cycles, and assessment checkpoints are constructed. The reviewed studies indicate that curriculum design is increasingly influenced by adaptive analytics, real-time feedback, learner modelling, and recommendation systems. However, the evidence also shows that dynamic curriculum systems require careful pedagogical governance to avoid reducing curriculum to algorithmic optimisation. Curriculum is therefore examined as a potentially dynamic construct whilst maintaining a distinction between demonstrated applications and broader conceptual implications.

### 5.1 Curriculum as a dynamic algorithmic construct

A central feature of post-linear curriculum is the movement from curriculum as a static document towards curriculum as a dynamic, data-informed process. This involves using mathematical and computational models to generate, evaluate, and revise learning pathways whilst remaining accountable to pedagogical aims. Early work, such as that by Guts et al. (2021), laid important groundwork by framing curriculum management as an optimisation problem. Their research presented mathematical models for tailoring university work plans based on concrete constraints such as student cohort characteristics and available classroom resources, treating the curriculum as a system that can be algorithmically adjusted for institutional efficiency. This concept extends beyond administrative optimisation into personalisation in modern AI systems. Chu and Ashraf (2025) show how AI leverages predictive analytics and natural language processing to evaluate individual student performance and propose unique learning pathways. Similarly, automatic curriculum learning from machine learning research, where task complexity is gradually increased based on an agent’s performance, offers a useful but incomplete parallel for structuring human learning in a nonlinear fashion. Chu and Ashraf (2025) reported a course completion rate of 89.72% and a retention rate of 91.44%, suggesting that carefully designed adaptive sequencing can support participation and persistence in specific contexts. These metrics are not general proof of educational transformation, because completion and retention do not capture all dimensions of learning quality, disciplinary understanding, equity, or agency. They nevertheless show that curriculum can function as a responsive structure built from data, algorithms, pedagogical rules, and human oversight rather than as a single pre-planned sequence.

### 5.2 Real-time adaptation to learner cognition

The dynamism of the post-linear curriculum is most visible in systems that adapt to learners’ cognitive and affective states. This moves beyond periodic personalisation to a more continuous modulation of the learning experience based on physiological and behavioural signals. Research by Zhu et al. (2024) demonstrates the potential of this approach, finding that an adaptive microlearning system significantly reduced unnecessary cognitive load caused by poor instructional design and improved learning adaptability compared with a conventional system. This effect was linked to the system’s ability to tailor content delivery, feedback, and learning paths in real time. A more granular example is provided by Chaouachi et al. (2019), who developed a system that adapts instructional strategy by monitoring learners’ electroencephalogram signals. By extracting real-time indexes for cognitive load and mental engagement, their system could dynamically switch between problem-solving tasks and worked examples to support more appropriate learning conditions. These findings support the possibility that real-time cognitive evaluation can influence learning outcomes and the emotional experience of learning. They also highlight important boundary conditions: physiological data raise privacy and consent concerns, cognitive-load optimisation may privilege measurable states over broader educational aims, and such systems require strong teacher oversight to avoid treating learners as data objects within a cybernetic feedback loop.

### 5.3 From predefined knowledge structures towards emergent knowledge processes

A dynamically adapting curriculum also invites a careful reconceptualisation of knowledge. In a rigid linear system, knowledge is often treated as a fixed body of content that exists before and independent of the learning process. In a post-linear paradigm, some knowledge remains disciplinary and cumulative, but the pathways through which learners encounter, connect, test, and extend that knowledge can become more emergent. Tomé and Gromova (2021), in their analysis of business education during the COVID-19 crisis, argue that complex and novel challenges reduce the sufficiency of stable knowledge stocks and create a need for strategies focused on knowledge exploration in situations marked by uncertainty. In such contexts, curriculum cannot simply deliver known answers; it needs to support inquiry, interpretation, and the generation of new understanding. This aligns with systems such as the Cross Domain Adaptive Recommendation System proposed by Shahbazi et al. (2025), which constructs continuously updated learning charts by integrating real-time user behaviour into evolving knowledge graphs. The Explainable Adaptive Learning module is especially relevant because it makes the logic of emergent pathways more transparent. In educational terms, the destination is not always a fixed point on a map but a horizon that shifts as learners investigate problems and receive contextual scaffolding. The curriculum’s role is therefore to provide a rich, responsive, and intelligible environment in which disciplinary knowledge, learner inquiry, system feedback, and teacher judgement interact. This interpretation shifts the purpose of AI-mediated curriculum from simple knowledge transfer towards knowledge cultivation within a complex, interactive system, whilst retaining the need for disciplinary structure, epistemic accountability, and human pedagogical judgement.

The evidence in this domain supports a tiered interpretation rather than a single claim of wholesale curricular transformation. Direct support is strongest for bounded mechanisms such as adaptive sequencing, real-time feedback, personalised recommendation, embedded assessment, and data-informed modification of learning pathways within recognisable curricular structures. The evidence that entire curricula become continuously emergent, or that educational systems routinely operate without predefined sequences, is substantially thinner and is concentrated in conceptual, design-oriented, or early-stage work. The 46 sources mapped to this domain therefore indicate the breadth of scholarship relevant to curriculum adaptation and emergent knowledge processes; they should not be read as 46 independent demonstrations of fully realised post-linear pedagogy. In this review, post-linear pedagogy is consequently treated as an integrative proposition about how sequencing can become more recursive and responsive under particular conditions, not as a claim that sequence, disciplinary progression, or teacher-designed curriculum should disappear. This distinction also separates evidence from speculation: adaptive features already documented in existing systems constitute the empirical base, whereas the extension from those features to a broader post-linear architecture remains a synthesis-derived hypothesis requiring longitudinal testing across subjects, educational levels, languages, and institutional settings. Table 2 is a conceptual orientation rather than an evidence map. The references associated with each row identify component-level precedents, adjacent mechanisms, or illustrative applications and should not be interpreted as direct empirical validation of every descriptor or of the complete named framework. Accordingly, evidential claims in this review are based on the narrative synthesis and corpus-level mapping reported above, whereas Table 2 is used only to compare conceptual relationships and future research directions.

**Table 2:** Emerging AI-education frameworks and key constructs.

Framework/Theory	Key constructs	Pedagogical implications	Technological enablers	Institutional adaptation mechanisms	Target learner profile	Future research priorities	Novelty/Potential impact	References
Adaptive epistemic ecosystem (AEE)	Human–AI co-evolution, emergent curricula, dynamic knowledge networks.	Teachers as epistemic designers; curricula adapt in real time.	Hybrid AI orchestration, adaptive dashboards.	Distributed governance, flexible policies.	All learners; highly variable cognitive profiles	Study co-adaptation dynamics, algorithmic learning pathways.	High: Systemic post-linear paradigm.	Hao et al. (2025); Leon et al. (2025); Mohtat and Khirfan (2023)
Nonlinear pedagogy model	Emergent pathways, interactionist learning, adaptive feedback	Supports flexible skill acquisition, responsive sequencing.	Learning analytics, predictive scaffolding	Curriculum redesign, teacher-as-facilitator	K-12 and tertiary learners	Examine long-term skill transfer and adaptive efficacy.	Moderate: Moves away from linear sequencing	Ertel et al. (2024); Ribeiro et al. (2021); Rudd et al. (2021)
Intelligent tutoring systems framework	Personalised scaffolding, real-time adaptive feedback.	Enables individualised learning; improves engagement.	Intelligent tutoring platforms, adaptive modules.	Teacher training for AI integration.	STEM learners, general learners	Optimise AI-human interaction, evaluate cognitive scaffolding.	High: Empirical evidence for co-adaptive learning.	Krygin et al. (2024); Sinatra (2024); Yilmaz et al. (2022)
Algorithmic epistemology	Knowledge validation, predictive assessment, automated reasoning.	Embeds assessment within learning, supports decentered authority.	Learning analytics, AI-driven evaluation.	Policy adaptation for AI assessment.	All learners, modular learning systems	Investigate bias, epistemic diversity, and assessment validity.	High: Challenges traditional authority in learning.	Gasparin and Schinckus (2022); Lumbanraja (2025); Maalsen (2023)
Gamification and AI-enhanced engagement	Motivation-driven, microlearning, adaptive feedback loops.	Increases persistence, engagement, and learning motivation.	Gamified platforms, AI performance trackers.	Adaptive classroom scheduling, incentive mechanisms.	Learners with variable engagement levels	Study motivational optimisation in AI-mediated learning.	Moderate: Personalised engagement scaling.	Ahmed Dahri et al. (2025); Benzizoune (2024); Elmashhara et al. (2024)
Project-based AI learning	Interdisciplinary projects, AI guidance, emergent skills.	Promotes creativity, problem-solving, and collaboration.	Simulation environments, AI project mentors.	Teacher-as-mentor, collaborative governance	Learners in STEM/Arts	Evaluate team dynamics, AI-facilitated innovation.	High: Co-learning in hybrid human–AI teams.	Daniels et al. (2025); Yuna et al. (2025); Zheng et al. (2024)
Hybrid STEM mentorship model	Adaptive scaffolding, predictive modelling, learner autonomy.	Personalised skill development, mentor-AI co-guidance.	Virtual labs, adaptive tutors, analytics dashboards.	Mentorship-integrated AI programmes.	STEM-focused learners	Study AI-mediated mentoring efficacy.	High: Bridges practical skills and algorithmic guidance.	Chang et al. (2024); Chung and Siegel (2025); Ibrahim et al. (2021)
Continuous assessment and micro-credentials	Embedded checkpoints, modular certification, adaptive credentialing.	Integrates assessment within learning; supports mastery.	Micro-credential platforms, adaptive assessment engines	Policy redesign, continuous monitoring.	All learners, professional learners.	Explore long-term skill retention, credential validity.	High: Reframes assessment as adaptive and dynamic.	Charles et al. (2025); Surono (2024a, 2024b)
Ethical-first AI framework	Fairness, inclusivity, transparency, and responsible AI.	Guides AI curriculum design, safeguards equity.	AI fairness audit tools, privacy-preserving analytics	Ethics committees, policy oversight.	All learners.	Study AI ethics in scalable adaptive systems.	High: Aligns AI deployment with human-centred values.	Celik (2023); Holmes et al. (2022); Nguyen et al. (2023)
Data-driven personalised learning	Predictive pathways, analytics-informed adaptation.	Supports learner-specific interventions; early risk detection.	Learning analytics dashboards, attention monitoring	Data-informed policy; teacher-AI collaboration.	Diverse learners.	Evaluate predictive accuracy and intervention outcomes.	High: Maximises learning efficiency via AI.	Guo et al. (2023); Jiang et al. (2022); Xia et al. (2024)
Collaborative AI learning	Peer-to-peer interaction, hybrid cognition, AI-facilitated collaboration.	Enhances teamwork, social learning, and shared knowledge.	AI collaboration platforms, recommendation engines	Teacher coordination, collaborative governance.	Groups of learners.	Study AI facilitation of group dynamics.	Moderate: Improves collaboration whilst maintaining personalisation.	Kovari (2025); Wang H. et al. (2025); Ye et al. (2025)
Open-source AI learning networks	Shared curricula, collaborative platforms, adaptive access.	Promotes knowledge exchange, scalability, and co-creation.	Open-source platforms, global analytics dashboards	Institutional adoption of shared tools.	Global learners with variable access.	Investigate global co-creation dynamics.	High: Democratises adaptive learning.	Oreshin et al. (2020); Pan et al. (2025)
Lifelong learning adaptive framework	Continuous upskilling, professional reskilling, and modular learning.	Supports adult and professional learners.	Skill gap analysis tools, adaptive micro-modules	Institutional programmes for adults, flexible scheduling.	Adult learners, career-focused	Study AI-mediated reskilling efficacy.	High: Prepares workforce for continuous change.	Adaimi and Thomaz (2022); Ghnemat et al. (2022); Tsuda et al. (2020)
Creative arts AI integration	Generative creativity, interdisciplinary hybrid learning.	Encourages artistic experimentation and hybrid cognition.	Generative AI, digital studios, collaborative tools	Flexible curriculum design, mentorship.	Arts/design learners.	Investigate AI influence on creative skill development.	Moderate: Integrates AI into creativity with personalisation.	Bedir Erişti and Freedman (2024); Omran Zailuddin et al. (2024); Peláez-Sánchez et al. (2024)

## 6 Institutional futures and the reconfiguration of authority

Institutional authority constitutes a further domain of AI-mediated change. The preceding sections addressed how AI affects cognition, curriculum, and assessment; the present discussion examines how those changes alter teacher identity, institutional legitimacy, and accountability structures. This distinction reduces thematic overlap by separating pedagogical adaptation from organisational authority and governance.

Studies on teacher identity, institutional legitimacy, and algorithmic accountability show that institutional transformation is not a direct or uniform outcome of AI adoption. Authority is renegotiated unevenly across classroom practice, professional roles, policy frameworks, procurement decisions, and governance infrastructures. Three linked patterns are visible in the corpus: reformation of teacher identity in response to technological mediation, institutional struggles over legitimacy in AI-mediated education, and governance challenges surrounding algorithmic accountability.

The transformation of teacher identity is a frontline consequence of AI integration, requiring educators to navigate a shift from being primary knowledge authorities to becoming orchestrators of hybrid learning environments. The reviewed examples indicate that this is not a simple linear upgrade but a context-dependent and negotiated process. Studies of language teachers, such as the case of a Chinese language teacher in Australia analysed by Yang and Han (2022), reveal that identity transformation follows a cognitive trajectory of transformative learning. This process, driven by critical reflection, involves reconciling pre-existing pedagogical beliefs with new environmental demands. Similarly, a multimodal study of novice English teachers in China by Zhou et al. (2025) identified a non-linear progression through identity configurations: from initial compliance with traditional methods, through experimentation, to eventual dialogic enactment of student-centred pedagogy. This evolution is mediated by subtle, embodied strategies like gaze, gesture, and vocal modulation, underscoring that professional agency and identity are performed and negotiated in real-time classroom interactions. These cases suggest that changes in teacher identity are strongly conditioned by professional agency: their capacity to actively interpret, adapt to, and shape their new roles amidst institutional pressures. The transformation is therefore deeply personal and context-dependent, challenging any notion of a one-size-fits-all model for the “AI-ready teacher.”

The redefinition of the teacher’s role also raises questions about institutional legitimacy in AI-mediated learning. Traditional educational institutions have often grounded authority in human expertise and structured, human-centred procedural oversight. The growing use of AI in specific cognitive and diagnostic tasks can challenge some of these assumptions without eliminating the continuing need for accountable human oversight. Frimpong (2025) conceptualises this challenge as institutional expiration, a state not of operational failure but of epistemic misalignment. Expiration Theory proposes that institutions may become conceptually misaligned when foundational logics no longer fit environments shaped by algorithmic reasoning and decision-making. In this review, the practical relevance of that argument lies in the possible gap between policy ambition and institutional capacity, including technological infrastructure, organisational capability, governance support, and the ability to exercise meaningful human oversight. This is treated as an interpretive proposition rather than as an established cross-context effect. Maintaining legitimacy in AI-mediated education therefore requires more than technology procurement; it requires transparent reconsideration of authority, oversight, and institutional value whilst retaining accountable human judgement.

The governance challenges inherent in this new reality crystallise around the problem of algorithmic accountability. When AI systems make or inform decisions about student pathways, assessments, and resource allocation, they become de facto policy actors, yet their operations are often opaque and developed by private entities outside traditional democratic oversight. This creates what Wang (2024) identifies as a distinct set of governance challenges: the fast, iterative development of AI that outpaces policy cycles, the inherent lack of interpretability in complex models, and the unpredictable emergence of new capabilities. To address this, scholars propose moving beyond technical fixes towards robust, multi-layered governance frameworks. Oncioiu and Bularca (2025) demonstrate that effective governance is environmentally cultivated; their study found that institutional governance, when clearly developed from a genuine knowledge-sharing culture, significantly enhances students’ legal and ethical AI literacy. This suggests governance is not just a set of rules but an enabling environment that fosters critical engagement. For accountability to be meaningful, frameworks need clear responsibility assignments. Cech’s (2021) Algorithmic Accountability Agency Framework (A3 Framework) is instructive here, analysing accountability through the agency of both the actor (the AI system/user) and the forum (those holding them to account). This structured approach is vital for designing mechanisms where human oversight remains meaningful, ensuring that the delegation of tasks to algorithms does not equate to an abdication of ultimate human responsibility and ethical judgement.

**Figure 4:**
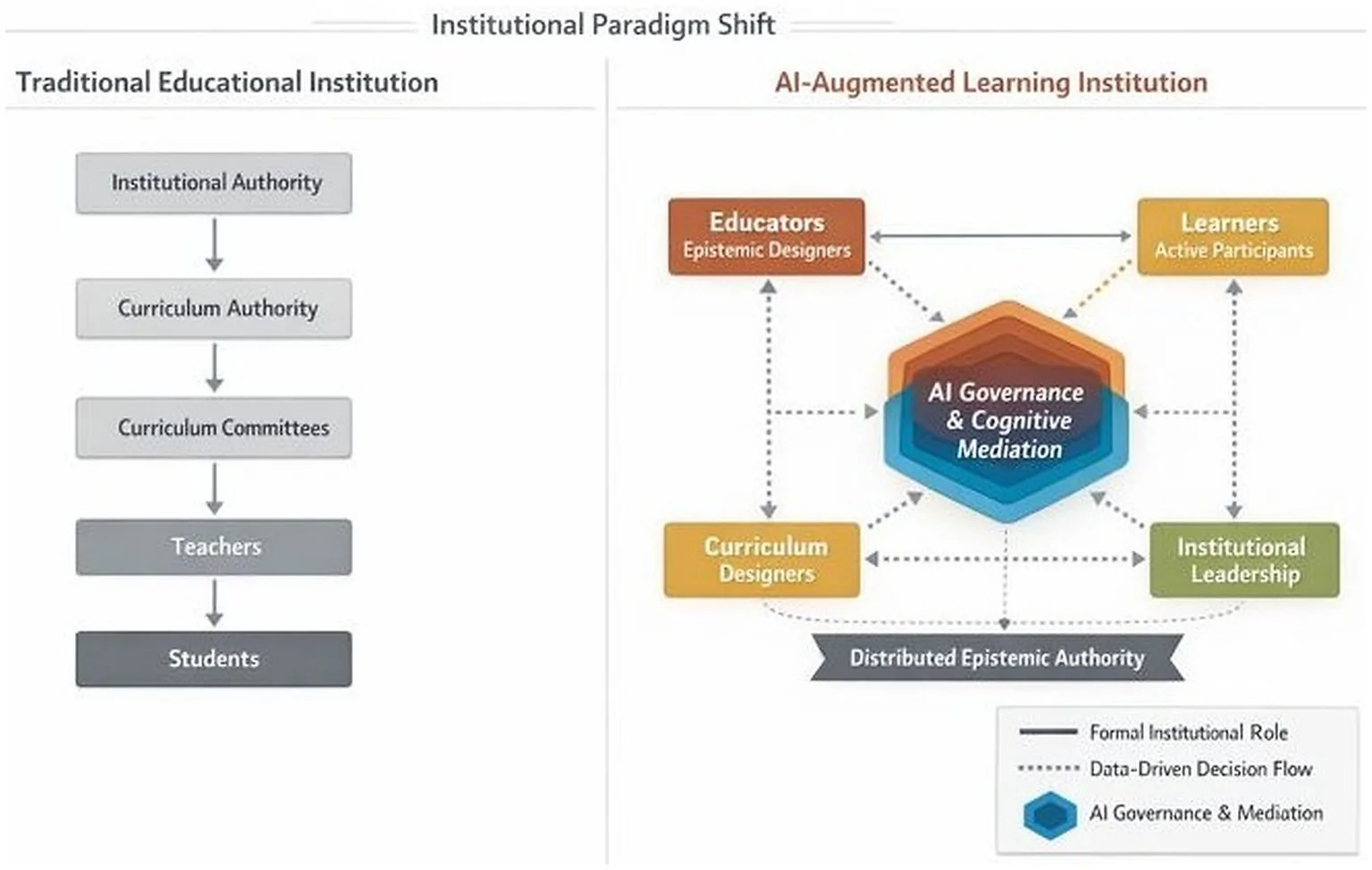
Institutional reconfiguration under AI governance.

The reconfiguration of authority in education links institutional viability to adaptive capacity, but this relationship is neither automatic nor purely technological. Teacher identity, institutional legitimacy, and algorithmic governance are interconnected strands of a single organisational challenge. Institutions that respond productively are likely to be those that treat AI neither as a neutral tool nor as an inevitable replacement for professional judgement, but as a catalyst for re-examining authority, accountability, curriculum, and public trust. This requires moving beyond narrow digital literacy initiatives towards institutional metacognition: the continuous capacity to examine and update the assumptions, values, and processes that define education itself. Contemporary studies suggest that failure may arise not only from technological obsolescence but also from conceptual inertia, weak governance, and insufficient participation by educators and learners. The path forward therefore depends on deliberate, ethical, and participatory redesign of the educational compact for an age of hybrid intelligence (Figure 4).

## 7 Global trajectories and divergent AI education futures

The reviewed literature indicates that the global integration of artificial intelligence into education is not following a singular or linear path. Instead, studies point to divergent trajectories shaped by infrastructure, policy capacity, pedagogical philosophy, language, culture, funding, and institutional readiness. UNESCO’s technology-in-education analysis emphasises that the educational value of technology depends on access, governance regulation, and teacher preparation rather than on availability alone (Global Education Monitoring Report Team, 2023). This position is consistent with the evidence reviewed here: AI can support learning under some conditions, but it can also deepen dependency, inequity, surveillance, and platform concentration when deployed without contextual safeguards. The following trajectories are therefore analytical patterns rather than mutually exclusive or universally observed models.

### 7.1 High-automation education systems

One trajectory identified within the limited global-trajectory subset concerns highly automated educational environments, particularly in contexts prioritising scalability and operational efficiency. This path is characterised by the deployment of technologies like social robots, intelligent tutoring systems, and automated administrative platforms aimed at streamlining or replacing human-led processes. A systematic review by Topali et al. (2025) of AI in K-12 settings found that a significant portion of implemented technologies, such as certain intelligent systems, focus narrowly on optimising learning outcomes, often at the expense of pedagogical depth and teacher involvement. This approach can lead to what the review identifies as a lack of “contextualization under concrete course conditions” and insufficient attention to “teacher agency and autonomy.” The drive towards full automation is also evident in higher education administration, as explored in the case of Makerere University Business School. Kayanja et al. (2025) document the push towards “fully automated and paperless systems,” highlighting the potential for enhanced efficiency and accessibility. However, this research simultaneously exposes critical bottlenecks: resistance to change, gaps in digital literacy, and resource constraints, particularly in developing regions. This evidence suggests that whilst high-automation systems offer clear operational benefits, their successful implementation and educational value are far from guaranteed. A primary risk is that an overemphasis on automation may instrumentalise education, reducing it to a standardised, data-driven process that marginalises the nuanced, relational, and context-dependent aspects of teaching and learning that are difficult to quantify.

### 7.2 Human-centred AI pedagogies

A contrasting set of studies and conceptual contributions examines human-centred approaches. This approach explicitly subordinates technology to pedagogical goals and human judgement, seeking to augment rather than replace educators. Le Dinh et al. (2025) formalise this in their proposed “Human-Centred AI for Systematic Literature Reviews (HCAI-SLR) framework,” which embeds human oversight and ethical checkpoints at every stage of AI-augmented research. This model illustrates a design position in which efficiency is pursued alongside rigour and ethical responsibility; it does not establish that this balance is achieved across educational settings. At the level of assessment, a core function where automation raises concerns about academic integrity, Balducci (2024) argues for a humanistic redesign. He proposes safeguarding learning by designing assessments anchored in “the use of skills and knowledge by real people in realistic situations,” with the aim of making them less susceptible to substitution by generative AI. This pedagogical philosophy reframes the problem from one of detecting AI use to one of designing for authentic human performance. These sources illustrate a conceptual contrast rather than an empirically established binary: some accounts emphasise system optimisation and scale, whereas human-centred accounts emphasise professional judgement, authentic performance, and pedagogical purpose.

### 7.3 Hybrid governance models

A third trajectory discussed in the reviewed literature concerns hybrid governance models intended to coordinate technological systems, institutional policy, and human agency. Within these accounts, governance is framed not only as top-down control but also as an institutional capacity for participation, coordination, and adaptation. The reviewed studies suggest that governance effectiveness is context-dependent and linked to institutional culture, implementation capacity, and clear responsibility. For instance, a large-scale diagnostic study across 81 Chinese universities by Zhao et al. (2025) revealed severe structural misalignments despite widespread policy adoption. They found a “high cognition, low practice” gap amongst faculty and a disconnect between student demand for AI skills and institutional supply. This case indicates that governance cannot be reduced to strategic plans; it includes concrete mechanisms for resource allocation, cross-disciplinary support, and practical faculty development. The governance problem identified in this strand is therefore one of translation and implementation. This supports further examination of models that connect high-level institutional strategy with grounded support for pedagogical change and capacity building, rather than assuming that policy adoption alone changes teaching practice or resource distribution.

### 7.4 Risks of epistemic inequality

A major implication of these global trajectories is the potential exacerbation of epistemic inequality. This refers not merely to unequal access to technology but to the systematic devaluation and exclusion of certain knowledge systems, perspectives, languages, and ways of knowing within AI-mediated education. Scholars argue that this constitutes a form of epistemic injustice. As Koch (2020) notes, discussions of responsible innovation often overlook how to make science internally more inclusive, a critique directly applicable to AI in education, where tool design and algorithmic training data can perpetuate biases. Mitova (2023) further socialises the concept of epistemic injustice, showing how it is rooted in social power relations. In AI-mediated education, this can manifest when systems trained on dominant languages, curricula, or cultural assumptions become arbiters of quality, relevance, or correctness. Decolonial analyses of AI ethics add that international governance templates may reproduce colonial epistemic hierarchies when they neglect plural moral traditions and local educational priorities (Nemorin, 2026). The resulting risk is not only a digital divide but an epistemic divide, in which some communities gain access to tools whilst losing control over the categories and values through which learning is interpreted.

The reviewed literature outlines several plausible but competing trajectories rather than a single forecast for global education. High-automation arrangements may increase efficiency and scale but can also create risks of de-professionalisation, reductive standardisation, surveillance, and encoded bias. Human-centred arrangements place greater weight on teacher judgement, participation, and equity, but their feasibility depends on professional development, institutional capacity, and governance. Hybrid arrangements combine automated and human decision-making but still require explicit accountability for contested decisions. These trajectories are analytical possibilities, not mutually exclusive predictions, and the limited evidence density in the global-trajectories domain does not support claims about which pathway will dominate. The central implication is therefore conditional: the educational consequences of AI depend on how technical capability is coupled with institutional authority, local context, epistemic diversity, and mechanisms for public and pedagogical accountability.

## 8 Ethical horizons and pedagogical risks

Limitations, counter-evidence, and pedagogical risks are central to the synthesis. As artificial intelligence moves from auxiliary tool to infrastructural component, its integration raises technical, ethical, epistemological, and institutional concerns. The discussion therefore moves beyond immediate issues of data privacy and bias to examine cognitive dependency, loss of epistemic diversity, and algorithmic normalisation of learning. These risks are not presented as inevitable outcomes. They are plausible trajectories that require empirical testing, participatory design, teacher oversight, and accountable institutional policy.

The risk of cognitive dependency emerges from the design of highly adaptive, personalised learning systems. Research demonstrates that algorithms can model student behaviour and optimise pathways with considerable precision. For instance, Li and Lu (2025) developed an intelligent system using a multimodal fusion algorithm and an adaptive learning module that achieved high precision (95.3%) in resource recommendation, significantly improving measured learning outcomes. Similarly, Xu (2025) found that AI-driven personalised teaching led to gains in student engagement and satisfaction. However, these efficiency gains create a pedagogical tension: learners may become accustomed to system-generated prompts, recommendations, explanations, and feedback, with fewer opportunities to practise independent problem framing, uncertainty management, and metacognitive regulation. The concern is not that AI assistance inevitably weakens cognition, but that poorly governed assistance may shift too much epistemic labour from learners and teachers to opaque systems. Cognitive dependency therefore functions as a design and governance problem rather than as an unavoidable consequence of AI use.

A second systemic risk is the loss of epistemic diversity. Epistemic diversity, the variety of ways of knowing, reasoning, and constructing knowledge across cultures, disciplines, languages, and cognitive styles, is a cornerstone of a robust intellectual ecosystem. Critical scholarship raises concern that globally deployed AI systems may favour and reproduce dominant, often Western, English-language, and neurotypical epistemic frameworks. Gobbo and Russo (2020) argue that the push towards a monolingual lingua franca in academia, often facilitated by English-medium instruction and AI tools trained on English-language corpora, can hinder epistemic diversity. Omodan (2023) and Perales et al. (2026) similarly show how indigenous, local, and multilingual knowledge systems remain vulnerable to marginalisation. UNESCO’s multilingual dissemination of technology-in-education debates illustrates the need for broader linguistic representation, but the review corpus still remains dominated by English-language scholarship (Global Education Monitoring Report Team, 2023). For adaptive epistemic ecosystems, diversity cannot be treated as an add-on. It functions as a design criterion across data sources, curriculum models, assessment criteria, language interfaces, and governance structures.

Algorithmic normalisation of learning provides a mechanism through which cognitive dependency and epistemic narrowing can become institutionalised. Advanced adaptive systems, such as the one proposed by Li and Lu (2025), which constructs a student-resource interaction graph, operate by identifying patterns, modelling preferred learning trajectories, and recommending efficient paths to predefined outcomes. This process can support standardisation and scale, but it also applies normative pressure by defining optimal progress, appropriate engagement, and correct understanding through aggregated data from past learners. Kohnke and Foung (2024) contextualise this risk within data colonialism in education, showing how data extraction and platform dependence can reproduce asymmetrical power relations. Critical data literacy research therefore becomes essential because learners and educators require the capacity to interrogate how categories, metrics, and recommendations are produced (Atenas et al., 2025). Without such capacity, adaptive systems may quietly normalise narrow forms of participation whilst presenting them as neutral personalisation.

The synthesis of these three risks reveals a coherent ethical horizon. Personalised efficiency can encourage cognitive dependency when learners rely on automated guidance without developing independent judgement. Data-intensive personalisation can produce algorithmic normalisation when systems define desirable learning pathways through historical patterns. Global deployment can narrow epistemic diversity when systems are trained and governed through limited linguistic, cultural, and institutional assumptions. Some recent scholarship moves from risk identification towards proactive design principles. For example, Baeyaert (2025) proposes reframing ethical AI design through the lens of neurodiversity, advocating adaptive fairness and participatory design. These principles align with the ecosystem framework because they treat learners as heterogeneous epistemic agents rather than as interchangeable data profiles. The synthesis therefore proposes learner autonomy, epistemic plurality, explainability, auditability, and teacher-mediated interpretation as safeguards to be evaluated in future implementations of adaptive epistemic ecosystems.

**Figure 5:**
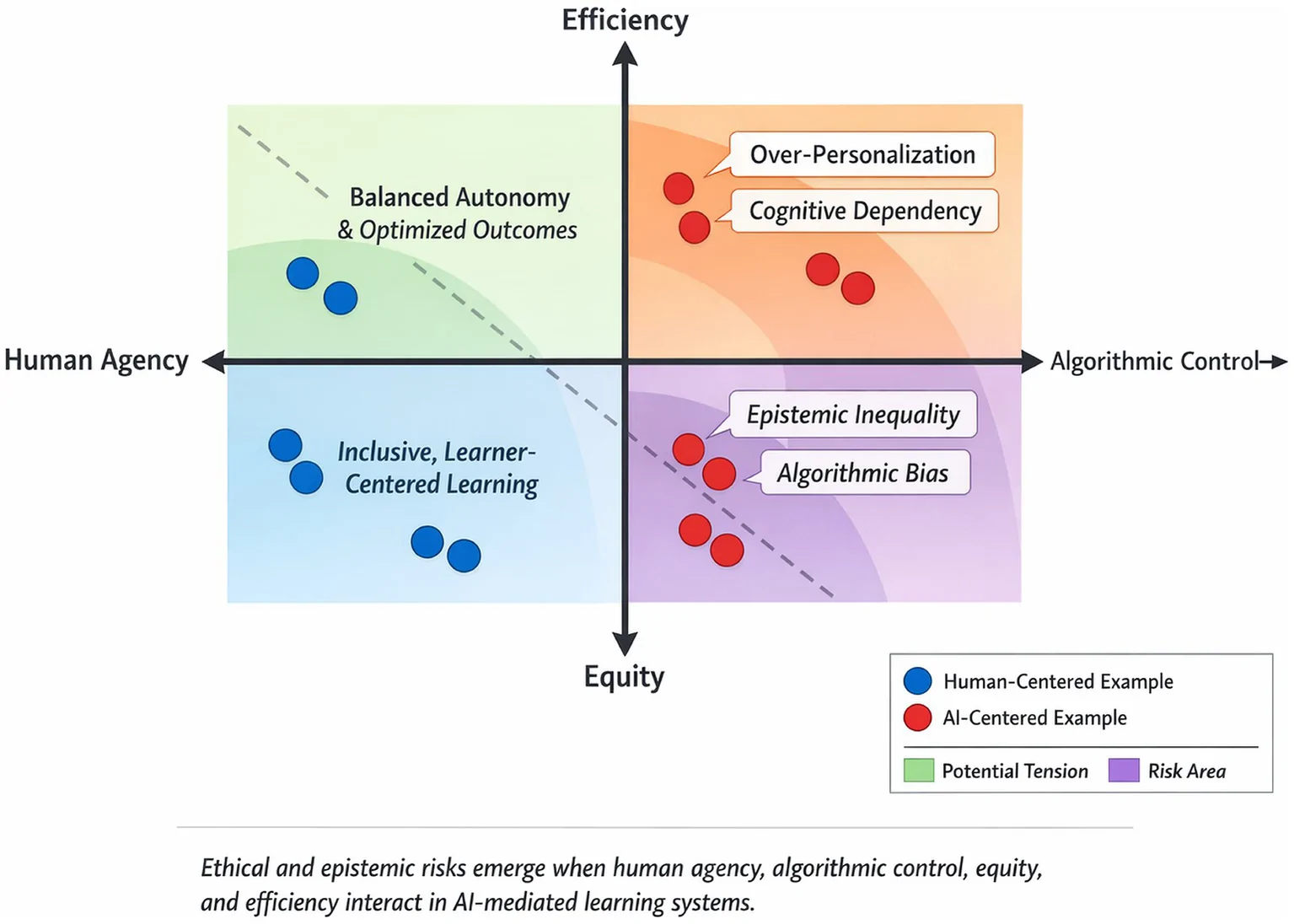
Ethical and epistemic tensions in AI-mediated learning.

The evidence therefore requires a more cautious interpretation than a simple transformation narrative allows. Studies reporting positive effects often evaluate bounded interventions, short time frames, specific platform functions, or narrow performance indicators. Critical studies emphasise implementation gaps, algorithmic opacity, teacher autonomy, platform governance, data colonialism, epistemic injustice, and uneven infrastructural capacity (Al-Zahrani, 2024; Galante et al., 2024; Holmes et al., 2022; Mbah and Jarvis, 2026; Topali et al., 2025; Yan et al., 2025). Additional critical scholarship on pandemic educational technology, decolonial AI ethics, and global technology governance reinforces this caution (Nemorin, 2026; Williamson et al., 2020). The central issue is not whether AI is beneficial or harmful in general, but under what pedagogical, cultural, institutional, and governance conditions particular AI systems expand or constrain educational capabilities. The adaptive epistemic ecosystem framework is valuable only if it remains open to negative evidence, implementation failure, and competing theoretical interpretations (Figure 5).

## 9 Forward research and policy agenda

**Figure 6:**
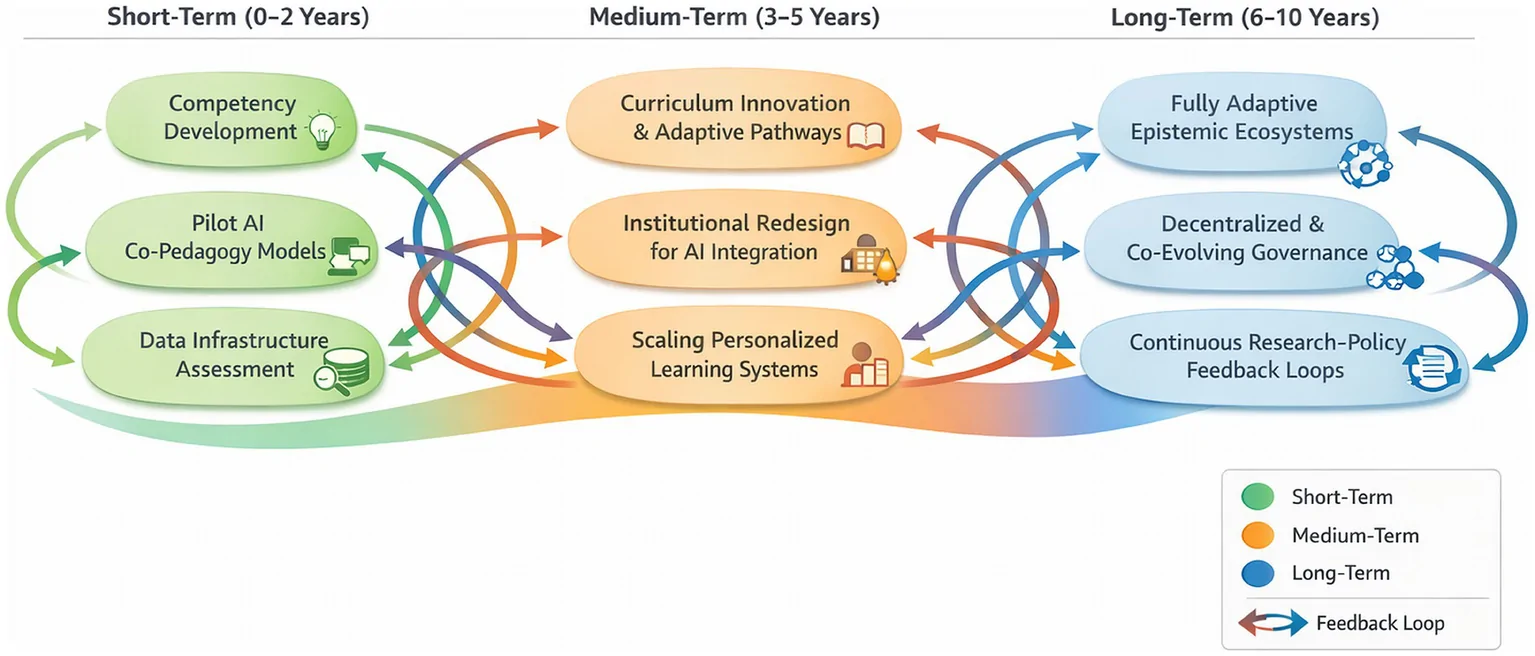
Forward-looking research and policy agenda.

The synthesis points to a research and policy agenda grounded in the gaps, cautions, and boundary conditions identified above. Sections 3 through 8 report evidence-grounded patterns and counter-evidence from the reviewed corpus; the statements in this section are forward-looking interpretations and research propositions derived from that synthesis and should not be read as findings already demonstrated across educational systems. The agenda is organised around three domains: research priorities for the next decade, institutional redesign priorities, and educator competency development. It does not assume that AI adoption itself produces educational improvement. Improvement is treated as conditional on evidence quality, pedagogical purpose, equity safeguards, public accountability, and institutional capacity. Figure 6 summarises the agenda by linking longitudinal evidence, governance redesign, curriculum adaptation, teacher professional learning, and epistemic justice.

### 9.1 Research priorities for the next decade

Future research needs to move beyond evaluating the efficiency of isolated AI tools and towards understanding the complex, systemic implications of embedding intelligence within educational structures. A primary priority is longitudinal and holistic analysis of AI integration. As highlighted by Zou et al. (2025), whilst trends such as AI and VR are well documented, stronger evidence is needed on sustained pedagogical effects across cognitive, relational, and affective domains, not only learning outcomes. This aligns with the critique that many current implementations lack a solid pedagogical foundation. A key research direction is therefore the development and validation of models that assess human-AI co-adaptation, teacher mediation, learner autonomy, and institutional governance over time. Comparative studies across regions, languages, school types, and resource settings are also necessary because existing evidence is unevenly distributed. Research designs that include negative cases, implementation failures, teacher resistance, and learner harms would make the field more balanced and less dependent on successful pilot studies. Methodologically, mixed-method longitudinal studies, participatory design research, algorithm audits, and comparative policy analysis are especially important for testing the adaptive epistemic ecosystem framework as an empirical proposition rather than a rhetorical claim.

### 9.2 Institutional redesign priorities

Institutional redesign is necessary because AI-mediated learning cannot be governed effectively through procurement decisions alone. Several reviewed studies and policy analyses identify curriculum and programme modernisation as one institutional response to AI-mediated change. Mohiuddin et al. (2023), in analysing priorities for Saudi Arabia’s Vision 2030, identify the new modern curriculum and industry-based academic learning outcomes as top priorities for bridging the gap between education and labour-market needs. This requires moving beyond static disciplinary silos towards dynamic, modular, and interdisciplinary curricula that can adapt to changing knowledge and skill demands whilst preserving disciplinary depth. Concurrently, institutions need stronger governance structures for data protection, algorithmic accountability, explainability, procurement transparency, and teacher participation. The UNESCO technology-in-education report places governance regulation and teacher preparation alongside access as system-wide conditions for responsible educational technology use (Global Education Monitoring Report Team, 2023). In the context of the present review, AI governance is best treated by universities, schools, and ministries as part of educational quality assurance rather than as a separate technical compliance issue.

### 9.3 Educator competencies for AI-mediated education

The role of the educator is shifting from knowledge deliverer towards epistemic designer and cognitive mediator, although this shift varies by discipline, level, institutional culture, and resource context. Research on teacher educators in Vietnam (Nguyen, 2023) identifies a foundational framework comprising knowledge and skills, ethical manner, motivation, and self-reflection. Within this broader framework, several competencies become especially important for AI-mediated education. The first is orchestration and pedagogical design expertise: educators need the ability to decide when AI can support personalisation, feedback, or resource discovery, and when human-led dialogue, peer interaction, and disciplinary modelling are pedagogically preferable. The second is algorithmic and data literacy: educators need enough understanding of data sources, model limitations, bias, explainability, and privacy to interpret AI outputs critically. The third is ethical and relational judgement: educators need to protect learner autonomy, inclusion, care, and trust in environments where automated feedback can influence learner self-concept and progression. The fourth is epistemic facilitation: educators need to help learners evaluate, challenge, and contribute to knowledge rather than simply consume AI-generated outputs. These competencies position teachers not as displaced actors but as accountable professionals who mediate between learners, technologies, institutions, and communities.

Across the agenda, research, institutional action, and educator development are interdependent. Research on epistemic ecosystems can inform institutional curriculum redesign, which in turn defines the competency frameworks for educator preparation. A significant bottleneck, visible in studies from diverse contexts, is the misalignment between rapid technological change and slow-moving institutional structures and professional development systems. Fragmented action risks exacerbating inequality, fostering educator resistance, and realising only a narrow portion of AI’s pedagogical potential. A systemic and synchronised approach is therefore needed: research generates robust evidence, institutions build accountable governance and adaptive curricula, and educators receive support as critical designers of human-AI learning environments. This agenda reframes the future of AI in education as a matter of public educational design rather than technological adoption alone.

## 10 Conclusion

This systematic narrative review synthesised 124 sources identified through a structured search covering January 2020 to January 2026 and formally mapped across the 2020–2025 evidence corpus to examine how artificial intelligence is reshaping educational systems beyond its role as an instructional tool. The review combines explicit eligibility criteria, staged screening, quality appraisal, structured extraction, inductive thematic synthesis, evidence mapping, citation auditing, and claim calibration. Across the corpus, AI is associated with changes in pedagogy, curriculum design, learning analytics, assessment, teacher roles, learner agency, institutional governance, and ethical debate, but the distribution and strength of evidence are uneven. The findings support a cautious interpretation of movement towards more adaptive and data-informed arrangements; they do not demonstrate that AI automatically transforms education or improves learning. Adaptive epistemic ecosystems and post-linear pedagogy are therefore advanced as synthesis-derived, integrative conceptual frameworks rather than universal empirical claims. Their contribution lies in specifying relationships amongst human-AI cognition, adaptive curriculum, learning-data infrastructures, epistemic validation, governance, institutional transformation, and boundary conditions whilst preserving explicit attention to power, language bias, inequality, teacher agency, and epistemic justice. Direct findings from the reviewed literature are distinguished from the authors’ integrative interpretation and from the future-oriented propositions generated by the framework. The evidence base remains constrained by English-language selection, concentration in recent and technologically affirmative scholarship, limited longitudinal research, and sparse documentation of failed or harmful implementations. The framework should therefore be tested, qualified, or rejected through longitudinal, comparative, multilingual, and context-sensitive research. The educational value of AI is most defensible where it strengthens human judgement, expands learner agency, protects epistemic diversity, and remains accountable to pedagogical and democratic purposes.

## References

[ref1] Adaimi R. Thomaz E. (2022). Lifelong adaptive machine learning for sensor-based human activity recognition using prototypical networks. Sensors 22:6881. doi: 10.3390/s22186881, 36146230 PMC9504213

[ref2] Ahmed Dahri N. Yahaya N. Mugahed Al-Rahmi W. Almuqren L. Almgren A. S. Alshimai A. . (2025). The effect of AI gamification on students’ engagement and academic achievement in Malaysia: SEM analysis perspectives. IEEE Access 13, 70791–70810. doi: 10.1109/ACCESS.2025.3560567

[ref3] Ajisoko P. (2020). The use of Duolingo apps to improve English vocabulary learning. Int. J. Emerg. Technol. Learn. 15:149. doi: 10.3991/ijet.v15i07.13229

[ref4] Akgun S. Greenhow C. (2022). Artificial intelligence in education: addressing ethical challenges in K-12 settings. AI Ethics 2, 431–440. doi: 10.1007/s43681-021-00096-7, 34790956 PMC8455229

[ref5] Akintola A. S. Akintayo M. Kadri T. Oforgu C. M. Michael M. Nwanna M. (2025). Adaptive AI systems in education: real-time personalised learning pathways for skill development. J. Art. Intell. Mach. Learn. Data Sci. 3, 2489–2494. doi: 10.51219/JAIMLD/Akinyemi-Sadeeq-Akintola/534

[ref6] Almoubayyed H. Fancsali S. E. Ritter S. (2023). Instruction-embedded assessment for reading ability in adaptive mathematics software LAK23: 13th International Learning Analytics and Knowledge Conference. New York, NY, USA: ACM. 366–377

[ref7] Alnaim M. Al-Otaibi G. (2025). Learning disabilities teachers’ perceptions of employing artificial intelligence applications in teaching their students. Int. J. Eval. Res. Educ. 14:2732. doi: 10.11591/ijere.v14i4.32888

[ref8] Al-Zahrani A. M. (2024). Unveiling the shadows: beyond the hype of AI in education. Heliyon 10:e30696. doi: 10.1016/j.heliyon.2024.e30696, 38737255 PMC11087970

[ref9] Atenas J. Havemann L. Nerantzi C. (2025). Critical and creative pedagogies for artificial intelligence and data literacy: an epistemic data justice approach for academic practice. Res. Learn. Technol. 32: 3296. doi: 10.25304/rlt.v32.3296

[ref10] Baeyaert J. (2025). The philosophy of cognitive diversity: rethinking ethical AI design through the lens of neurodiversity. Sustain. Futur. 10:101126. doi: 10.1016/j.sftr.2025.101126

[ref11] Balducci B. (2024). AI and student assessment in human-centered education. Front. Educ. 9: 1383148 doi: 10.3389/feduc.2024.1383148

[ref12] Bawa I. Bawa S. (2025). Bridging EdTech gaps: examining learning equity in low-income educational settings. Int. J. Educ. Dev. 118:103398. doi: 10.1016/j.ijedudev.2025.103398

[ref13] Baze C. L. González-Howard M. Sampson V. Crawford R. Hamilton X. (2025). “Okay we’re doing my idea”: how students enact epistemic agency and power in a design-based engineering context. Educ. Sci. 15:402. doi: 10.3390/educsci15040402

[ref14] Bazoukis G. A. Halkidis S. T. Pepes E. Venardos P. (2024). AI in education: pedagogical and ethical analysis of the implementation of ASSISTments in the school environment. Eur. J. Sci. Math. Educ. 12, 428–451. doi: 10.30935/scimath/14902

[ref15] Bedir Erişti S. D. Freedman K. (2024). Integrating digital technologies and AI in art education: pedagogical competencies and the evolution of digital visual culture. Particip. Educ. Res. 11: 5779. doi: 10.17275/per.24.94.11.6

[ref16] Belenguer L. (2022). AI bias: exploring discriminatory algorithmic decision-making models and the application of possible machine-centric solutions adapted from the pharmaceutical industry. AI Ethics 2, 771–787. doi: 10.1007/s43681-022-00138-8, 35194591 PMC8830968

[ref17] Benzizoune O. (2024). Enhancing classroom management and student engagement: the role of ClassDojo and gamification in education. J. Engl. Lang. Teach. Appl. Linguist. 6, 106–114. doi: 10.32996/jeltal.2024.6.3.13

[ref18] Bergamaschi Ganapini M. Campbell M. Fabiano F. Horesh L. Lenchner J. Loreggia A. . (2025). Fast, slow, and metacognitive thinking in AI. NPJ Artif. Intell. 1:27. doi: 10.1038/s44387-025-00027-5

[ref19] Bromilow L. Milne N. Woods C. T. Dowsett C. K. Keogh J. W. L. (2025). The effectiveness of linear and nonlinear pedagogical approaches in team-invasion ball sports: a systematic review. Sports Med. Open 11:90. doi: 10.1186/s40798-025-00893-y, 40759833 PMC12321719

[ref20] Camara W. J. Mattern K. (2022). Inflection point: the role of testing in admissions decisions in a postpandemic environment. Educ. Meas. Issues Pract. 41, 10–15. doi: 10.1111/emip.12493

[ref21] Cech F. (2021). The agency of the forum: mechanisms for algorithmic accountability through the lens of agency. J. Responsible Technol. 7–8:100015. doi: 10.1016/j.jrt.2021.100015

[ref22] Celik I. (2023). Towards intelligent-TPACK: an empirical study on teachers’ professional knowledge to ethically integrate artificial intelligence (AI)-based tools into education. Comput. Hum. Behav. 138:107468. doi: 10.1016/j.chb.2022.107468

[ref23] Chan C. K. Y. (2023). A comprehensive AI policy education framework for university teaching and learning. Int. J. Educ. Technol. High. Educ. 20:38. doi: 10.1186/s41239-023-00408-3

[ref24] Chang C.-N. Hui J. Justus-Smith C. Wang T.-W. (2024). Navigating STEM careers with AI mentors: a new IDP journey. Front. Art. Intell. 7: 1461137 doi: 10.3389/frai.2024.1461137, 39439844 PMC11493768

[ref25] Chaouachi M. Jraidi I. Lajoie S. P. Frasson C. (2019). Enhancing the learning experience using real-time cognitive evaluation. Int. J. Inf. Educ. Technol. 9, 678–688. doi: 10.18178/ijiet.2019.9.10.1287

[ref26] Charles T. Zoelfakar S. Sebastian S. (2025). “Micro-credentials in vocational and professional training,” in Integrating Micro-Credentials with AI in Open Education Durak G.and Çankaya S. (Hershey, PA, USA: IGI Global Scientific Publishing), 217–250. doi: 10.4018/979-8-3693-5488-9.ch010

[ref27] Chatzichristofis S. A. Tsopozidis A. Kyriakidou-Zacharoudiou A. Evripidou S. Amanatiadis A. (2025). Designing an AI-supported framework for literary text adaptation in primary classrooms. AI 6:150. doi: 10.3390/ai6070150

[ref28] Chen B. C. (2025). Using Vygotsky’s sociocultural theory to explore ethnic cultural representation in Taiwanese preschool children’s play. Front. Educ. 10: 1569322. doi: 10.3389/feduc.2025.1569322

[ref29] Christensen G. (2024). Three concepts of power: Foucault, Bourdieu, and Habermas. Power Educ. 16, 182–195. doi: 10.1177/17577438231187129

[ref30] Chu T. S. Ashraf M. (2025). Artificial intelligence in curriculum design: a data-driven approach to higher education innovation. Knowledge 5:14. doi: 10.3390/knowledge5030014

[ref31] Chung C.-J. Siegel J. (2025). Impact of the COVID-19 Pandemic on Robofest: A Longitudinal Analysis of STEM Education Outcomes. 2025 IEEE integrated STEM education conference (ISEC), Princeton, NJ, USA. 1–8.

[ref32] Conklin T. A. (2016). Knewton (an adaptive learning platform available at https://www.knewton.com/). Acad. Manag. Learn. Educ. 15, 635–639. doi: 10.5465/amle.2016.0206

[ref33] Corbett F. Spinello E. (2020). Connectivism and leadership: harnessing a learning theory for the digital age to redefine leadership in the twenty-first century. Heliyon 6:e03250. doi: 10.1016/j.heliyon.2020.e03250, 31993523 PMC6971348

[ref34] Cui W. Xue Z. Thai K. P. (2018). Performance Comparison of An AI-based Adaptive Learning System in China. Xi'an, China: 2018 Chinese Automation Congress (CAC).

[ref35] Damaševičius R. (2025). Reconceptualizing education in the stakeholder era: a futures perspective. Sustainable Futures 10:101515. doi: 10.1016/j.sftr.2025.101515

[ref36] Daniels M. Kelly É. Flynn S. Kelly J. (2025). Advancing project leadership education through AI-enhanced game-based learning. Project Leadership Soc. 6:100189. doi: 10.1016/j.plas.2025.100189

[ref37] Dutta S. Ranjan S. Mishra S. Sharma V. Hewage P. Iwendi C. (2024). Enhancing educational adaptability: a review and analysis of AI-driven adaptive learning platforms In: 2024 4th International Conference on Innovative Practices in Technology and Management (ICIPTM) 1–5 Piscataway, NJ, USA: IEEE. doi: 10.1109/ICIPTM59628.2024.10563448

[ref38] Elmashhara M. G. De Cicco R. Silva S. C. Hammerschmidt M. Silva M. L. (2024). How gamifying AI shapes customer motivation, engagement, and purchase behaviour. Psychol. Mark. 41, 134–150. doi: 10.1002/mar.21912

[ref39] English A. R. (2023). “Dewey, existential uncertainty and non-affirmative democratic education,” in Educational Governance Research:(Cham, Switzerland: Springer).

[ref40] Ertel M. Monsma E. Brian A. (2024). Developing hot executive functioning skills and autonomous motivation in soccer through a nonlinear pedagogical approach in secondary physical education. J. Phys. Educ. Recreat. Dance 95, 33–40. doi: 10.1080/07303084.2023.2269211

[ref41] Fang Y. Ren Z. Hu X. Graesser A. C. (2019). A meta-analysis of the effectiveness of ALEKS on learning. Educ. Psychol. 39, 1278–1292. doi: 10.1080/01443410.2018.1495829

[ref42] Farrow R. (2023). The possibilities and limits of explicable artificial intelligence (XAI) in education: a socio-technical perspective. Learn. Media Technol. 48, 266–279. doi: 10.1080/17439884.2023.2185630

[ref43] Feng M. Huang C. Collins K. (2023). “Promising long term effects of ASSISTments online math homework support,” in Communications in Computer and Information Science, (Cham, Switzerland: Springer), 212–217.

[ref44] Foster M. E. (2024). Evaluating the impact of supplemental computer-assisted math instruction in elementary school: a conceptual replication. J. Res. Educ. Eff. 17, 94–118. doi: 10.1080/19345747.2023.2174919

[ref45] Frimpong V. (2025). When institutions cannot keep up with artificial intelligence: expiration theory and the risk of institutional invalidation. Adm. Sci. 15:263. doi: 10.3390/admsci15070263

[ref46] Galante A. Piccardo E. Marcel F. Zeaiter L. F. dela Cruz J. W. N. Barise A. (2024). Decolonizing language learning in digital environments through the voices of plurilingual learners in the global south. Appl. Linguist. 47, 273–292. doi: 10.1093/applin/amae090

[ref47] Garzón J. Patiño E. Marulanda C. (2025). Systematic review of artificial intelligence in education: trends, benefits, and challenges. Multimodal Technol. Interact. 9:84. doi: 10.3390/mti9080084

[ref48] Gasparin M. Schinckus C. (2022). The performativity of algorithmic trading: the epistemology of flash crashes. Knowl. Cult. 10:104. doi: 10.22381/kc10120226

[ref49] Ghnemat R. Shaout A. Al-Sowi A. M. (2022). Higher education transformation for artificial intelligence revolution: transformation framework. Int. J. Emerg. Technol. Learn. 17, 224–241. doi: 10.3991/ijet.v17i19.33309

[ref50] Global Education Monitoring Report Team (2023). Global Education Monitoring Report 2023: Technology in Education: A Tool on Whose Terms? Paris, France: GEM Report UNESCO.

[ref51] Gobbo F. Russo F. (2020). Epistemic diversity and the question of lingua franca in science and philosophy. Found. Sci. 25, 185–207. doi: 10.1007/s10699-019-09631-6

[ref52] Grammatikos N. A. Anagnostopoulou E. Apostolou D. Mentzas G. (2025). “An AI-powered learning companion for adaptive and personalized STEM education,” in Lecture Notes in Computer Science, (Cham, Switzerland: Springer), 87–95.

[ref53] Guo X. He X. Pei Z. (2023). Data-driven Personalized Learning Proceedings of the 2023 6th International Conference on Educational Technology Management, New York, NY, USA: ACM. 49–54.

[ref54] Guts A. K. Senkovskaya A. A. Furayeva I. I. (2021). Modelling and construction of curriculum optimization algorithms in order to improve the effectiveness of management of the educational process. J. Phys. Conf. Ser. 1791:012074. doi: 10.1088/1742-6596/1791/1/012074, 42288387

[ref55] Guzmán-Valenzuela C. Chiappa R. Gómez-González C. (2025). Transforming regional higher education: the decolonising role of indigenous-inspired universities in Latin America. Int. J. Educ. Res. 133:102731. doi: 10.1016/j.ijer.2025.102731

[ref56] Hanna M. G. Pantanowitz L. Jackson B. Palmer O. Visweswaran S. Pantanowitz J. . (2025). Ethical and Bias considerations in artificial intelligence/machine learning. Mod. Pathol. 38:100686. doi: 10.1016/j.modpat.2024.100686, 39694331

[ref57] Hao X. Demir E. Eyers D. (2025). Beyond human-in-the-loop: sensemaking between artificial intelligence and human intelligence collaboration. Sustain. Futur. 10: 101152. doi: 10.1016/j.sftr.2025.101152

[ref58] Harris D. B. Roter D. L. (2024). Profound love and dialogue: Paulo Freire and liberation education. Health Literacy Res. Pract. 8: e118e120. doi: 10.3928/24748307-20240613-02, 38979815 PMC11230641

[ref59] Holmes W. Porayska-Pomsta K. Holstein K. Sutherland E. Baker T. Shum S. B. . (2022). Ethics of AI in education: towards a community-wide framework. Int. J. Artif. Intell. Educ. 32, 504–526. doi: 10.1007/s40593-021-00239-1

[ref60] Hossain K. I. (2024). Reviewing the role of culture in English language learning: challenges and opportunities for educators. Soc. Sci. Hum. Open 9:100781. doi: 10.1016/j.ssaho.2023.100781

[ref61] Hummel S. (2025). Ethical and responsible AI in education: situated ethics for democratic learning. Educ. Sci. 15:1594. doi: 10.3390/educsci15121594

[ref62] Ibrahim A. A. Panda S. Adya G. (2021). High School STEM Clubs in a Virtual World 2021 IEEE Integrated STEM Education Conference (ISEC), Piscataway, NJ, USA: IEEE. 225–225.

[ref63] Jaldemark J. Lundin J. Säljö R. Edwards J. Gegenfurtner A. Holmes W. . (2025). A multidisciplinary research agenda for artificial intelligence, education, learning, and instruction. Postdigit. Sci. Educ. 7, 1414–1450. doi: 10.1007/s42438-025-00602-8

[ref64] Javadinejad A. Davari M. (2026). The architecture of AI-mediated learning: a three-layer framework. Appl. Sci. 16:4991. doi: 10.3390/app16104991

[ref65] Jiang B. Li X. Yang S. Kong Y. Cheng W. Hao C. . (2022). Data-driven personalized learning path planning based on cognitive diagnostic assessments in MOOCs. Appl. Sci. 12:3982. doi: 10.3390/app12083982

[ref66] Kayanja W. Kyambade M. Kiggundu T. (2025). Exploring digital transformation in higher education setting: the shift to fully automated and paperless systems. Cogent Educ. 12: 2489800. doi: 10.1080/2331186X.2025.2489800

[ref67] Kehinde B. (2023). Supporting STEM success: digital curriculum innovations to foster student retention. Int. J. Sci. Res. Manage. 11, 982–1000. doi: 10.18535/ijsrm/v11i04.ec3

[ref68] Knox J. (2020). Artificial intelligence and education in China. Learn. Media Technol. 45, 298–311. doi: 10.1080/17439884.2020.1754236

[ref69] Koch S. (2020). Responsible research, inequality in science and epistemic injustice: an attempt to open up thinking about inclusiveness in the context of RI/RRI. J. Responsible Innov. 7, 672–679. doi: 10.1080/23299460.2020.1780094

[ref70] Kohnke L. Foung D. (2024). Deconstructing the normalization of data colonialism in educational technology. Educ. Sci. 14:57. doi: 10.3390/educsci14010057

[ref71] Kovari A. (2025). A systematic review of AI-powered collaborative learning in higher education: trends and outcomes from the last decade. Soc. Sci. Humanit. Open 11:101335. doi: 10.1016/j.ssaho.2025.101335

[ref72] Krygin A. I. Gumerov M. R. Moskalenko N. A. Sychev O. A. (2024). A framework for developing intelligent tutoring systems based on domain models in the form of decision trees. Pattern Recognit. Image Anal. 34, 710–716. doi: 10.1134/S1054661824700561

[ref73] Kuchynska I. Orlov O. Kobysia A. Kutsak L. Yesselbayeva A. (2025). Adaptation of pedagogical approaches to the implementation of intelligent systems in the educational process. Periodicals Eng. Nat. Sci. 13, 281–294. doi: 10.21533/pen.v13.i1.299

[ref74] Kumor T. Uribe-Flórez L. Trespalacios J. Yang D. (2024). ALEKS in high school mathematics classrooms: exploring teachers’ perceptions and use of this tool. TechTrends 68, 506–519. doi: 10.1007/s11528-024-00955-0

[ref75] Le Dinh T. Le T. D. Uwizeyemungu S. Pelletier C. (2025). Human-centered artificial intelligence in higher education: a framework for systematic literature reviews. Information 16:240. doi: 10.3390/info16030240

[ref76] Lehtinen A. Kostiainen E. Näykki P. (2023). Co-construction of knowledge and socioemotional interaction in pre-service teachers’ video-based online collaborative learning. Teach. Teach. Educ. 133:104299. doi: 10.1016/j.tate.2023.104299

[ref77] Leon C. Lipuma J. Oviedo-Torres X. (2025). Artificial intelligence in STEM education: a transdisciplinary framework for engagement and innovation. Front. Educ. 10: 1619888. doi: 10.3389/feduc.2025.1619888

[ref78] Li Y. Lu B. (2025). Intelligent educational systems based on adaptive learning algorithms and multimodal behaviour modeling. PeerJ Comput. Sci. 11:e3157. doi: 10.7717/peerj-cs.3157, 40989423 PMC12453766

[ref79] Linden K. Pemberton L. A. Webster L. (2019). Evaluating the Bones of Adaptive Learning: Do the Initial Promises Really Increase Student Engagement and Flexible Learning Within First Year Anatomy Subjects? Valencia, Spain: Editorial Universitat Politècnica de València.

[ref80] Liu Y. Fu Z. (2024). Hybrid intelligence: design for sustainable multiverse via integrative cognitive creation model through human–computer collaboration. Appl. Sci. 14:4662. doi: 10.3390/app14114662

[ref81] Lu H. Limniou M. Zhang X. (2025). Metacognition and social presence in connectivist learning: an analysis of Bilibili interactions. Educ. Sci. 15:1673. doi: 10.3390/educsci15121673

[ref82] Lumbanraja A. D. (2025). Beyond the human subject: reconceptualizing cyberlaw through virtual ontology and algorithmic epistemology in the web 3.0 era. Int. J. Semiot. Law Rev. Int. Sémiot. Jurid. 39, 1639–1658. doi: 10.1007/s11196-025-10376-8

[ref83] Maalsen S. (2023). Algorithmic epistemologies and methodologies: algorithmic harm, algorithmic care and situated algorithmic knowledges. Prog. Hum. Geogr. 47, 197–214. doi: 10.1177/03091325221149439

[ref84] Maass K. Sorge S. Romero-Ariza M. Hesse A. Straser O. (2022). Promoting active citizenship in mathematics and science teaching. Int. J. Sci. Math. Educ. 20, 727–746. doi: 10.1007/s10763-021-10182-1, 34177402 PMC8214369

[ref85] Makar K. Fry K. English L. (2023). Primary students’ learning about citizenship through data science. ZDM 55, 967–979. doi: 10.1007/s11858-022-01450-7, 36619684 PMC9806803

[ref86] Mamlok D. (2025). Landscapes of sociotechnical imaginaries in education: a theoretical examination of integrating artificial intelligence in education. Found. Sci. 30, 529–540. doi: 10.1007/s10699-024-09948-x

[ref87] Mbah M. F. Jarvis L. (2026). Towards a decolonial AI sovereignty model for indigenous educational settings. Cogent Educ. 13: 2626631. doi: 10.1080/2331186X.2026.2626631

[ref88] Mengliyev I. Meylikulov S. Fayzullayeva Z. Kobulova M. (2024). Education Artificial Intelligence Systems and Their Use in Teaching, AIP Conference Proceedings, Melville, NY, USA: AIP Publishing. 3244: 030079.

[ref89] Mirzaei S. Nikmehr H. Liu S. Marmolejo-Ramos F. (2025). Creativity in learning analytics: a systematic literature review. J. Intelligence 13:153. doi: 10.3390/jintelligence13120153, 41440814 PMC12733823

[ref90] Mishara P. (2024). The ethical implications of AI in education: privacy, bias, and accountability. J. Informatics Educ. Res. 4: 35503556. doi: 10.52783/jier.v4i2.1827

[ref91] Mitova V. (2023). Socialising epistemic risk: on the risks of epistemic injustice. Metaphilosophy 54, 539–552. doi: 10.1111/meta.12640

[ref92] Mohammad Bagheri M. (2015). Intelligent and adaptive tutoring systems: how to integrate learners. Int. J. Educ. 7:1. doi: 10.5296/ije.v7i2.7079

[ref93] Mohiuddin K. Nasr O. A. Nadhmi Miladi M. Fatima H. Shahwar S. Noorulhasan Naveed Q. (2023). Potentialities and priorities for higher educational development in Saudi Arabia for the next decade: critical reflections of the vision 2030 framework. Heliyon 9:e16368. doi: 10.1016/j.heliyon.2023.e16368, 37251831 PMC10220240

[ref94] Mohtat N. Khirfan L. (2023). Epistemic justice in flood-adaptive green infrastructure planning: the recognition of local experiential knowledge in Thorncliffe Park, Toronto. Landsc. Urban Plan. 238:104834. doi: 10.1016/j.landurbplan.2023.104834

[ref95] Molenaar I. (2022). The concept of hybrid human-AI regulation: exemplifying how to support young learners’ self-regulated learning. Comput. Educ. Art. Intell. 3:100070. doi: 10.1016/j.caeai.2022.100070, 42574925

[ref96] Munna M. S. H. Hossain M. R. Saylo K. R. (2024). Digital education revolution: evaluating LMS-based learning and traditional approaches. J. Innov. Technol. Converg. 6, 21–40. doi: 10.69478/JITC2024v6n002a03

[ref97] Nemorin S. (2026). Towards decolonising the ethics of AI in education. Global. Soc. Educ. 24, 1260–1272. doi: 10.1080/14767724.2024.2333821

[ref98] Nguyen N. T. L. (2023). How to develop four competencies for teacher educators. Front. Educ. 8:1147143. doi: 10.3389/feduc.2023.1147143

[ref99] Nguyen A. Ngo H. N. Hong Y. Dang B. Nguyen B.-P. T. (2023). Ethical principles for artificial intelligence in education. Educ. Inf. Technol. 28, 4221–4241. doi: 10.1007/s10639-022-11316-w, 36254344 PMC9558020

[ref100] Nieminen J. H. Haataja E. Cobb P. J. (2025). From active learners to knowledge contributors: authentic assessment as a catalyst for students’ epistemic agency. Teach. High. Educ. 30, 970–990. doi: 10.1080/13562517.2024.2332252

[ref101] Nieminen J. H. Ketonen L. (2024). Epistemic agency: a link between assessment, knowledge and society. High. Educ. 88, 777–794. doi: 10.1007/s10734-023-01142-5

[ref102] Norberg K. A. Almoubayyed H. De Ley L. Murphy A. Weldon K. Ritter S. (2025). Rewriting content with GPT-4 to support emerging readers in adaptive mathematics software. Int. J. Artif. Intell. Educ. 35, 587–626. doi: 10.1007/s40593-024-00420-2

[ref103] Nugraha A. E. Wibowo D. Hendrawan B. (2024). Paulo Freire’s critical pedagogy analysis of educational transformation. Majority Sci. J. 2, 220–228. doi: 10.61942/msj.v2i2.157

[ref104] Nwobodo R. E. E. (2025). Freire’s problem posing concept of education: a model for Nigeria system of education. J. Educ. Rev. Provision 5, 64–75. doi: 10.55885/jerp.v5i1.322

[ref105] Omodan B. I. (2023). Unveiling epistemic injustice in education: a critical analysis of alternative approaches. Soc. Sci. Hum. Open 8:100699. doi: 10.1016/j.ssaho.2023.100699

[ref106] Omodan B. I. (2025). The roles of epistemology and decoloniality in addressing power dynamics in university education. Global. Soc. Educ. 23, 1226–1240. doi: 10.1080/14767724.2024.2335661

[ref107] Omran Zailuddin M. F. N. Nik Harun N. A. Abdul Rahim H. A. Kamaruzaman A. F. Berahim M. H. Harun M. H. . (2024). Redefining creative education: a case study analysis of AI in design courses. J. Res. Innov. Teach. Learn. 17, 282–296. doi: 10.1108/JRIT-01-2024-0019

[ref108] Oncioiu I. Bularca A. R. (2025). Artificial intelligence governance in higher education: the role of knowledge-based strategies in fostering legal awareness and ethical artificial intelligence literacy. Societies 15:144. doi: 10.3390/soc15060144

[ref109] Oreshin S. Filchenkov A. Petrusha P. Krasheninnikov E. Panfilov A. Glukhov I. . (2020). Implementing a Machine Learning Approach to Predicting Students Academic Outcomes ACM International Conference Proceeding Series. New York, NY, USA: ACM.

[ref110] Ouyang Z. Jiang Y. Liu H. (2024). The effects of Duolingo, an AI-integrated technology, on EFL learners’ willingness to communicate and engagement in online classes. Int. Rev. Res. Open Distrib. Learn. 25, 97–115. doi: 10.19173/irrodl.v25i3.7677

[ref111] Pan J. Zhao Z. Han D. (2025). Academic performance prediction using machine learning approaches: a survey. IEEE Trans. Learn. Technol. 18, 351–368. doi: 10.1109/TLT.2025.3554174

[ref112] Peláez-Sánchez I. C. Velarde-Camaqui D. Glasserman-Morales L. D. (2024). The impact of large language models on higher education: exploring the connection between AI and education 4.0. Front. Educ. 9: 1392091. doi: 10.3389/feduc.2024.1392091

[ref113] Perales W. F. Ulla M. B. Busbus S. O. Bolintao J. J. M. Montederamos H. H. (2026). Decolonizing higher education institutions’ language curriculum: a collaborative autoethnography. Soc. Sci. Humanit. Open 13:102525. doi: 10.1016/j.ssaho.2026.102525

[ref114] Povey H. Adams G. (2021). Disordering mathematics, citizenship and socio-political research in mathematics education amongst the “rubble of words”. Res. Math. Educ. 23, 306–322. doi: 10.1080/14794802.2021.1994452

[ref115] Punziano G. (2025). Adaptive epistemology: embracing generative AI as a paradigm shift in social science. Societies 15:205. doi: 10.3390/soc15070205

[ref116] Quay J. (2019). “John Dewey’s conceptualisation of experience,” in Experiential Learning and Outdoor Education, (Abingdon, UK: Routledge), 71–90.

[ref117] Ribeiro J. Davids K. Silva P. Coutinho P. Barreira D. Garganta J. (2021). Talent development in sport requires athlete enrichment: contemporary insights from a nonlinear pedagogy and the athletic skills model. Sports Med. 51, 1115–1122. doi: 10.1007/s40279-021-01437-6, 33675517

[ref118] Rigopouli K. Kotsifakos D. Psaromiligkos Y. (2025). Vygotsky’s creativity options and ideas in 21st-century technology-enhanced learning design. Educ. Sci. 15:257. doi: 10.3390/educsci15020257

[ref119] Rudd J. Renshaw I. Savelsbergh G. J. P. Chow J. Y. Roberts W. Newcombe D. . (2021). Nonlinear Pedagogy and the Athletic Skills Model. The Importance of Play in Supporting Physical Literacy. New York, NY, USA: Routledge. doi: 10.4324/9781003025375, 33675517

[ref120] Sahakyan H. Gevorgyan A. . (2025). From disciplinary societies to algorithmic control: rethinking Foucault’s human subject in the digital age. Philosophies 10:73. doi: 10.3390/philosophies10040073

[ref121] Shah R. Cardozo M. L. Hjarrand J. (2024). Learning as ecosystems: shifting paradigms for more holistic programming in education and displacement. Int. J. Educ. Dev. 104:102943. doi: 10.1016/j.ijedudev.2023.102943

[ref122] Shahbazi Z. Jalali R. Shahbazi Z. (2025). Enhancing recommendation systems with real-time adaptive learning and multi-domain knowledge graphs. Big Data Cogn. Comput. 9:124. doi: 10.3390/bdcc9050124

[ref123] Shashkova N. Chetyrbok P. Lukyanova Y. Kazak A. Sidin M. (2025). “Artificial intelligence in education and training,” in Lecture Notes in Networks and Systems, (Cham, Switzerland: Springer), 365–371.

[ref124] Shortt M. Tilak S. Kuznetcova I. Martens B. Akinkuolie B. (2023). Gamification in mobile-assisted language learning: a systematic review of Duolingo literature from public release of 2012 to early 2020. Comput. Assist. Lang. Learn. 36, 517–554. doi: 10.1080/09588221.2021.1933540

[ref125] Sidji M. Smith W. Rogerson M. J. (2024). Adopting the Theory of Distributed Cognition for Human-AI Cooperation Proceedings of the 36th Australasian Conference on Human-Computer Interaction, New York, NY, USA: ACM. 774–779.

[ref126] Sinatra A. (2024). Usability heuristic review of an intelligent tutoring system framework. Human Factors Simulat. 139, 106113. doi: 10.54941/ahfe1005029

[ref127] Sun S. Else-Quest N. M. Hodges L. C. French A. M. Dowling R. (2021). The effects of ALEKS on mathematics learning in K-12 and higher education: a meta-analysis. Investig. Math. Learn. 13, 182–196. doi: 10.1080/19477503.2021.1926194

[ref128] Surono S. (2024a). Enhancing the excellence of quality management system auditors: a competency-based training model with instructional design strategic approaches and measurable outcomes. Devotion J. Res. Community Ser. 5, 949–960. doi: 10.59188/devotion.v5i8.781

[ref129] Surono S. (2024b). Standard of micro-credential quality management system to enhance employability and competitiveness of graduates. Asian J. Soc. Humanit. 3, 196–209. doi: 10.59888/ajosh.v3i1.433

[ref130] Toha M. A. (2026). Artificial intelligence, sustainability and higher education: a systematic literature review toward sustainable higher education (SHE) framework. Int. J. Educ. Manag. 40, 29–52. doi: 10.1108/IJEM-12-2024-0773, 35579975

[ref131] Tomé E. Gromova E. (2021). Development of emergent knowledge strategies and new dynamic capabilities for business education in a time of crisis. Sustainability 13:4518. doi: 10.3390/su13084518

[ref132] Tomisu H. Ueda J. Yamanaka T. (2025). The cognitive mirror: a framework for AI-powered metacognition and self-regulated learning. Front. Educ. 10: 1697554 doi: 10.3389/feduc.2025.1697554

[ref133] Topali P. Haelermans C. Molenaar I. Segers E. (2025). Pedagogical considerations in the automation era: a systematic literature review of AIEd in K-12 authentic settings. Br. Educ. Res. J. 51, 2777–2809. doi: 10.1002/berj.4200

[ref134] Tran M. Balasooriya C. Semmler C. Rhee J. (2025). Generative artificial intelligence: the ‘more knowledgeable other’ in a social constructivist framework of medical education. Npj Digital Medicine 8:430. doi: 10.1038/s41746-025-01823-8, 40646156 PMC12254308

[ref135] Tsuda B. Tye K. M. Siegelmann H. T. Sejnowski T. J. (2020). A modeling framework for adaptive lifelong learning with transfer and savings through gating in the prefrontal cortex. Proc. Natl. Acad. Sci. 117, 29872–29882. doi: 10.1073/pnas.2009591117, 33154155 PMC7703668

[ref136] Villegas-Ch W. Buenano-Fernandez D. Navarro A. M. Mera-Navarrete A. (2025). Adaptive intelligent tutoring systems for STEM education: analysis of the learning impact and effectiveness of personalized feedback. Smart Learn. Environ. 12:41. doi: 10.1186/s40561-025-00389-y

[ref137] Walker P. B. Haase J. J. Mehalick M. L. Steele C. T. Russell D. W. Davidson I. N. (2025). Harnessing metacognition for safe and responsible AI. Technologies 13:107. doi: 10.3390/technologies13030107

[ref138] Wang Y. (2024). Algorithmic decisions in education governance: implications and challenges. Discov. Educ. 3:229. doi: 10.1007/s44217-024-00337-x

[ref139] Wang Y. (2025). Artificial intelligence in student management systems to enhance academic performance monitoring and intervention. Sci. Rep. 15:35122. doi: 10.1038/s41598-025-19159-4, 41062725 PMC12508167

[ref140] Wang L. Liang G. (2025). Data-driven teaching and learning effect evaluation in online education. Int. J. Web-Based Learn. Teach. Technol. 20, 1–24. doi: 10.4018/IJWLTT.381307

[ref141] Wang H. Wang C. Chen Z. Liu F. Bao C. Xu X. (2025). Impact of AI-agent-supported collaborative learning on the learning outcomes of university programming courses. Educ. Inf. Technol. 30, 17717–17749. doi: 10.1007/s10639-025-13487-8

[ref142] Wang Y. Wang J. Zhang H. Song J. (2025b). Bridging prediction and decision: advances and challenges in data-driven optimization. Nexus 2:100057. doi: 10.1016/j.ynexs.2025.100057

[ref143] Wang Y. Zhang L. Li N. Wang C. Wang L. (2025a). How Chinese citizens understand the inflection point of COVID-19 pandemic: insight into formal mathematics and street mathematics. Front. Educ. 10: 1559007. doi: 10.3389/feduc.2025.1559007

[ref144] Wea D. Sri Pudjiarti E. Kristian Sarang R. (2023). Assessing the quality of learning materials in a learning management system (LMS): its impact on learning outcomes. Revista de Educación. 402, 51–64. doi: 10.59671/GC23O

[ref145] Williamson B. Eynon R. Potter J. (2020). Pandemic politics, pedagogies and practices: digital technologies and distance education during the coronavirus emergency. Learn. Media Technol. 45, 107–114. doi: 10.1080/17439884.2020.1761641

[ref146] Xia Y. Shin S.-Y. Shin K.-S. (2024). Designing personalized learning paths for foreign language acquisition using big data: theoretical and empirical analysis. Appl. Sci. 14:9506. doi: 10.3390/app14209506

[ref147] Xu X. (2025). AI optimization algorithms enhance higher education management and personalized teaching through empirical analysis. Sci. Rep. 15:10157. doi: 10.1038/s41598-025-94481-5, 40128297 PMC11933401

[ref148] Xue Y. Khalid F. (2026). Investigating the impact of metacognitive regulation on students’ academic performance: a quantitative study of Chinese distance learners. Front. Psychol. 17: 1726956. doi: 10.3389/fpsyg.2026.1726956, 41788270 PMC12957146

[ref149] Yan Y. Liu H. Chau T. (2025). A systematic review of AI ethics in education. J. Glob. Inf. Manag. 33, 1–50. doi: 10.4018/JGIM.386381

[ref150] Yang S. Han J. (2022). Perspectives of transformative learning and professional agency: a native Chinese language teacher’s story of teacher identity transformation in Australia. Front. Psychol. 13: 949673 doi: 10.3389/fpsyg.2022.949673, 36072040 PMC9443839

[ref151] Ye J. Yu Y. Zhang M. Long S. (2025). Synergizing AI and collaborative learning: a new approach to Chinese character acquisition in lower elementary education. Interact. Learn. Environ. 34, 697–1716. doi: 10.1080/10494820.2025.2528096

[ref152] Yilmaz R. Yurdugül H. Karaoğlan Yilmaz F. G. Şahi̇n M. Sulak S. Aydin F. . (2022). Smart MOOC integrated with intelligent tutoring: a system architecture and framework model proposal. Comput. Educ. Art. Intell. 3:100092. doi: 10.1016/j.caeai.2022.100092

[ref153] Yue M. Jong M. S. Y. Dai Y. (2022). Pedagogical design of K-12 artificial intelligence education: a systematic review. Sustainability 14:15620. doi: 10.3390/su142315620

[ref154] Yuna J. Minho K. Jiwon H. Jiwon L. Suyitno S. (2025). The role of the teacher as a facilitator in project-based learning with AI support. Al-Hijr J. Adulearn World 4, 36–46. doi: 10.55849/alhijr.v4i1.855

[ref155] Zawacki-Richter O. Marín V. I. Bond M. Gouverneur F. (2019). Systematic review of research on artificial intelligence applications in higher education – where are the educators? Int. J. Educ. Technol. High. Educ. 16:39. doi: 10.1186/s41239-019-0171-0

[ref156] Zhao T. Lin C. Qian C. Zhang X. (2025). Empowering teaching in higher education through artificial intelligence: a multidimensional exploration. Sustainability 18:147. doi: 10.3390/su18010147

[ref157] Zheng C. Yuan K. Guo B. Hadi Mogavi R. Peng Z. Ma S. . (2024). Charting the Future of AI in Project-Based Learning: A Co-design Exploration with Students Proceedings of the CHI Conference on Human Factors in Computing Systems, New York, NY, USA: ACM. 1–19.

[ref158] Zhou J. Li C. Cheng Y. (2025). Transforming pedagogical practices and teacher identity through multimodal (inter)action analysis: a case study of novice EFL teachers in China. Behav. Sci. 15:1050. doi: 10.3390/bs15081050, 40867407 PMC12383010

[ref159] Zhu B. Chau K. T. Mokmin N. A. M. (2024). Optimizing cognitive load and learning adaptability with adaptive microlearning for in-service personnel. Sci. Rep. 14:25960. doi: 10.1038/s41598-024-77122-1, 39472703 PMC11522668

[ref160] Zou Y. Kuek F. Feng W. Cheng X. (2025). Digital learning in the 21st century: trends, challenges, and innovations in technology integration. Front. Educ. 10: 1562391 doi: 10.3389/feduc.2025.1562391

